# Exploring the association between metabolic syndrome, its components and subsequent cancer incidence: A cohort study in Catalonia

**DOI:** 10.1002/cam4.7400

**Published:** 2024-08-16

**Authors:** Tomàs López‐Jiménez, Oleguer Plana‐Ripoll, Talita Duarte‐Salles, Martina Recalde, Matthew Bennett, Francesc Xavier‐Cos, Diana Puente

**Affiliations:** ^1^ Fundació Institut Universitari Per a La Recerca a L'Atenció Primària de Salut Jordi Gol I Gurina (IDIAPJGol) Barcelona Spain; ^2^ Universitat Autònoma de Barcelona, Bellaterra (Cerdanyola de Vallès) Barcelona Spain; ^3^ Programa de Doctorat en Metodologia de la Recerca Biomèdica i Salut Pública Universitat Autònoma de Barcelona Bellaterra (Cerdanyola del Vallès) Spain; ^4^ Department of Clinical Medicine Aarhus University Aarhus Denmark; ^5^ Department of Clinical Epidemiology Aarhus University and Aarhus University Hospital Aarhus Denmark; ^6^ International Agency for Research on Cancer (IARC‐WHO) Lyon Cedex France; ^7^ Department of Medical Informatics Erasmus University Medical Center Rotterdam The Netherlands; ^8^ DAP‐Cat Group, Unitat de Suport a la Recerca Barcelona Fundació Institut Universitari per a la recerca a l'Atenció Primària de Salut Jordi Gol i Gurina (IDIAPJGol) Barcelona Spain; ^9^ Innovation Office at Institut Català de la Salut Barcelona Spain

**Keywords:** cancer risk factors, epidemiology, metabolic studies, registries

## Abstract

**Background:**

Metabolic syndrome (MS) has emerged as a significant global health concern. The relationship between MS and the risk of cancer doesn‘t seem clear, whether examining by components or in combination. The objective of this study is to examine the relationship between MS, its components, and the overall risk of cancer, including the risk of 13 specific cancer types.

**Methods:**

We included 3,918,781 individuals aged 40 years or older sourced from the SIDIAP database between 2008 and 2017. Cox models were employed with MS components and their combinations. A subsample was created using a matched cohort (by age and sex). Incidence curves were computed to determine the time elapsed between the date of having 1–5 MS components and cancer incidence, compared to matched participants with no MS components, which showed that individuals who had one MS component experienced a greater incidence of cancer over 5 and 10 years than individuals with no MS, and the incidence rose with an increase in the number of MS components.

**Results:**

Individuals exposed to MS components were diagnosed with cancer earlier than those who were not exposed to them. In the Cox model, HDL (HR 1.46, 95% CI: 1.41–1.52) and Glycemia (HR 1.40, 95% CI: 1.37–1.44) were the individual combinations with the highest risk of overall cancer. In combinations with two components, the highest HR was HDL+Glycemia (HR 1.52, 95% CI: 1.45–1.59) and Glycemia+HBP (HR 1.48, 95% CI: 1.45–1.50). In combinations with three components, the highest HR was HDL+Glycemia+HBP (HR 1.58, 95% CI: 1.55–1.62).

**Conclusion:**

In summary, having one or more MS components raises the risk of developing at least 11 cancer types and these risk differ according to type of component included. Some sex differences are also observed. Our findings suggest that implementing prevention measures aimed at specific MS components may lower the risk of various cancer types.

## 1 INTRODUCTION

Metabolic Syndrome (MS) is characterized by a collection of cardiometabolic risk factors that commonly co‐exist. These factors include obesity, hypertension, dyslipidemia, and insulin resistance.^1^ The prevalence of MS in adults in Europe is about 10%–30%. In USA, the prevalence of MS was 32.5% in the 2011–2012 and increased to 36.9% in 2015–2016.^2^ The prevalence of MS represents a significant public health concern, and with rising rates of overweight and obesity anticipated in the future, the number of individuals affected by MS is projected to increase further.^3^ Although MS was in the beginning regarded as a likelihood for diabetes and cardiovascular disease,^4^ recent articles, including one from our group,^5^ have also identified a relationship among MS and various types of cancer.^6^, ^7^, ^8^

The fundamental pathophysiological mechanism linking MS and cancer remains poorly comprehended. Separate factors of MS such as obesity,^9^, ^10^ elevate glucose levels and hypertension^11^, ^12^ have been linked independently to an increased risk of various cancer types. Nevertheless, prior research has determined that the simultaneous presence of obesity and insulin resistance may constitute the primary factors underlying the link between MS and cancer.^6^, ^13^, ^14^

Obesity and others poor metabolic status, such as MS, have risen significantly in recent times,^15^ and these conditions are correlated with contemporary Western lifestyles. In a previous study,^5^ we observed an association between MS and different cancers utilizing relative risks, though absolute risks were not explored. Additionally, few studies have investigated the connection between the individual components of MS and their combinations with the risk of a wide numbers of cancers.^16^, ^17^, ^18^ Only one of these studies was population‐based, with a large database. There is a need to provide new evidence based on a large sample and focusing on the combination of individual MS components, a range of different cancer types, and using both relative and absolute measures of association. In our previous study,^5^ odds ratios were employed to assess the relationship between MS components and various types of cancer. Nevertheless, the objective of this present study is to expand our understanding by incorporating hazard ratios (HR) alongside absolute measures of association. While relative measures, such as HR, provide insights into the temporal aspects of the association, absolute measures, such as risk differences or absolute risks, allow us to quantify the direct impact of these relationships on the overall risk in the population. This dual approach not only enhances the comprehensiveness of our analysis but also ensures a more robust and nuanced interpretation of the intricate links between MS components and diverse cancer types among different populations.

The aim of this study is thus to examine the relationship among MS, its components, and the likelihood of developing overall cancer, along with the risk of 13 specific types of cancer. Both relative and absolute measures of association will be utilized to ensure a comprehensive understanding of these relationships.

## 2 MATERIALS AND METHODS

### 2.1 Design

We conducted a cohort study using data collected prospectively from the Information System for Research in Primary Care (SIDIAP; www.sidiap.org).

### 2.2 Data Source and setting

Data were collected from the SIDIAP database, covering the period from January 1, 2006, to December 31, 2017, which consists of the electronic health records of 286 primary healthcare centers (approximately 5.8 million individuals, 75% of the residents of Catalonia, Spain).^19^ The SIDIAP contains information on sociodemographic characteristics and clinical diagnoses, coded using the International Classification of Diseases, 10th revision (ICD‐10), clinical parameters, laboratory tests outcomes and prescriptions and dispensations of medicines (using the Anatomical Therapeutic Chemical (*ATC*) Classification *System*).^20^

### 2.3 Study population

We included all participants aged 40 years or older who did not have a prior cancer diagnosis as of the index date, which was either January 1, 2008, or the date when a participant turned 40 years of age if they were younger than 40 on January 1, 2008. We followed participants starting from the index date until they were diagnosed with their first incident (primary) cancer, until their death, until they were no longer part of the SIDIAP database, until they reached the age of 100 years, or until the conclusion of the study period (December 31, 2017). There was no minimum follow‐up time.

Patients were excluded from participation if two different cancer types were registered on the same date, if they presented with secondary cancers and metastases, if men were diagnosed with breast cancer, or there was a possible error in diagnosis (men with endometrial cancer or women with prostate cancer).

Information regarding the study's structure and the accuracy of the initial measurements has been documented in other publications.^5^, ^21^, ^22^

### 2.4 Cancer definition

Cases of cancer were identified as individuals diagnosed with cancer for the first time between January 1, 2008, and December 31, 2017. We included all cancers registered in the database (ICD‐10 C00‐C99). However, when the analysis was stratified by cancer type, the following cancers were included: colorectal (C18+C20), prostate (C61), liver (C22), bladder (C67), endometrial (C54), pancreas (C25), breast (C50), lung (C34), kidney (C64), thyroid (C73), Hodgkin lymphoma (C81), non‐Hodgkin lymphoma (C82–85), leukemia (C91–95) and other cancers (C00–C99 not included in the above definitions). We excluded benign tumors and tumors with uncertain or unknown behavior (D01–D48). The analyses for breast and endometrial cancer were stratified according to menopausal status (pre‐ and post‐), guided by evidence suggesting different effects of obesity and estrogens during these two life stages.^23^

### 2.5 MS definition

As per criteria set by the American Heart Association/National Heart, Lung, and Blood Institute (AHA/NHLBI), a diagnosis of MS is established in a patient if they exhibit three or more of the following components: Obesity, high blood pressure (HBP), reduced HDL cholesterol, elevated Triglycerides (TG) and high Glycemia.^1^ We characterized obesity as a body mass index (BMI) exceeding 30 kg/m^2^, serving as a measure of overall adiposity. Details of the definition of MS have been published elsewhere.^21^ In cases where multiple measurements for a component were documented on the same day, we calculated the mean of these values.

MS was treated as time‐varying factor.^24^ Individuals were considered unexposed until presenting with at least one MS component. Then, they were considered exposed to one component until presenting a second component. If they presented a second component, they were considered exposed to two components until presenting a third component, and so on.

Based on these definitions, we generated a combined variable ranging from 0 to 5 MS components, representing the presence of MS components.

### 2.6 Covariables

We also extracted information on age, sex, nationality (Spanish, non‐Spanish), the MEDEA deprivation index^25^ (a census tract‐based deprivation index used as a proxy of socioeconomic status in urban areas), smoking status, alcohol intake calculated in standard units, dispensation of drugs such as hormonal replacement therapy in menopausal women (ATC G03), paracetamol, aspirin, ibuprofen, presence of hepatitis and menopause (ICD‐10 N95). For women lacking data regarding menopausal status, we considered being ≥50 years of age as indicative of post‐menopausal status. For covariates with missing data, a “missing” response category was created.

### 2.7 Statistical analysis

We conducted a descriptive analysis of the baseline population, utilizing the mean (standard deviation) and median (percentile 25‐percertile 75) for numerical variables and percentages for categorical variables, stratified by the number of MS components.

A subsample was created using a matched cohort. In this subsample, a descriptive analysis and cumulative incidence curves were computed to determine the time elapsed between the date of having at least one of the MS components prior to cancer incidence, compared to age‐ and sex‐matched participants with no MS components. Sex matching was exact, while age matching had an interval of plus/minus 90 days. Each MS case was paired with a participant without MS components at the time of the pairing.

In the general cohort sample, we fitted Cox proportional hazards models with age as the underlined time scale to estimate hazard ratios (HR) and 95% confidence intervals (CI) for the association of the numbers of MS components, the combinations of different MS components, and individual numbers of MS components in relation to later cancer incidence, calculated separately for each cancer type. The cumulative incidence probability of cancer was estimated using the Aalen–Johansen estimator, which takes competing risks into account. Individuals with cancer contributed time to the study from their index date until their cancer diagnosis, whereas individuals without cancer contributed time to the study from their index date until the conclusion of follow‐up, date of death, or date of exit from the study, whichever occurred first. All models were adjusted for the MEDEA Deprivation Index, smoking status (never, former, current), alcohol intake (no consumption, low consumption, high consumption, and nationality Spanish, others). Hepatitis (B, C, and other/unspecified hepatitis) and other liver diseases (alcoholic liver disease, toxic liver disease, inflammatory liver disease, liver disease, unspecified, cystic liver disease) were accounted as a covariable in the analysis of liver cancer. Sensitivity analyses were conducted by rerunning the analyses excluding cases with non‐melanoma skin cancer (C44).

All statistical analyses were conducted using the software packages Stata 17 (StataCorp LLC., College Station, Texas, USA) and R version 4.1.2.

## 3 RESULTS

This study included 3,918,781 participants, of whom 315,803 were diagnosed with cancer during the follow‐up. The distribution of individuals with cancer was as follows: 36,270 colorectal; 5751 liver; 5421 pancreatic; 37,954 breast (7802 pre‐menopausal and 29,819 post‐menopausal); 5381 endometrial (464 pre‐menopausal and 4914 post‐menopausal); 20,788 bladder; 6824 kidney; 30,839 prostate; 681 Hodgkin lymphoma; 3611 non‐Hodgkin lymphoma; 6950 leukemia; 20,376 lung, 2707 thyroid, and 132,250 other cancers. We excluded 333 men with breast cancer and 7 individuals with an error in the cancer diagnosis (three men with endometrial cancer and four women with prostate cancer).

Baseline characteristics of all individuals categorized by the count of MS components are summarized in Table 1. During the study period, 1,990,124 individuals were diagnosed with 1 MS, 1,542,436 individuals were diagnosed with 2 MS, 1,332,084 were diagnosed with 3 MS, 672,031 were diagnosed with 4 MS and 259,549 were diagnosed with 5 MS. On average, those without any MS components were younger (mean age 47.9). Additionally, participants with a greater number of MS components tended to be older (mean age 56.0, 59.6, 61.9, 63.2, and 64.2 years for those with 1, 2, 3, 4, and 5 MS components, respectively). Women accounted for 49.5% of participants with no components and 51.2%, 50.6%, 51.8%, 52.9%, and 57.5% in participants with 1 –5 MS components, respectively.

**TABLE 1:** Cohort characteristics by number of metabolic syndrome (MS) components.

	Metabolic syndrome^a^					
	0 components	1 component	2 components	3 components	4 component	5 components
*N* total Percentage	2,269,299 57.9%	1,990,124 50.8%	1,542,436 39.4%	1,100,966 28.1%	672,0.31 17.1%	259,549 6.6%
Age mean (SD)	47.9 (10.9)	56.0 (13.9)	59.6 (13.9)	61.9 (1367)	63.2 (13.1)	64.2 (12.5)
Median (IQR)	42.8 (40–52.3)	53.1 (44.1–64.8)	58.1 (47.9–69.7)	61.1 (50.9–72.1)	63.0 (53.1–73.2)	64.2 (54.8–73.7)
Sex						
Men, *n* (%)	1,145,512 (50.5)	970,198 (48.8)	762,562 (49.4)	541,410 (49.2)	316,761 (47.1)	110,318 (42.5)
Women, *n* (%)	1,123,787 (49.5)	1,019,926 (51.2)	779,874 (50.6)	559,556 (50.8)	355,270 (52.9)	149,231 (57.5)
Nationality						
Spanish	1,932,488 (85.2)	1,805,863 (90.7)	1,421,452 (92.2)	1,024,203 (93.0)	629,231 (93.6)	244,578 (94.2)
Non‐Spanish	336,811 (14.8)	184,261 (9.3)	120,984 (7.8)	76,763 (7.0)	42,800 (6.4)	14,971 (5.8)
MEDEA index						
Quintile 1	392,538 (17.1)	319,445 (16.1)	207,079 (13.4)	132,499 (12.0)	25,945 (10.0)	74,462 (11.1)
Quintile 2	336,583 (14.8)	297,495 14.9 ()	224,673 (14.6)	155,774 (14.2)	34,391 (13.3)	92,184 (13.7)
Quintile 3	318,352 (14.0)	286,013 (14.4)	228,228 (14.8)	164,245 (14.9)	39,047 (15.0)	100,035 (14.9)
Quintile 4	306,698 (13.5)	275,415 (13.8)	228,203 (14.8)	168,800 (15.3)	42,138 (16.2)	105,291 (15.7)
Quintile 5	292,090 (12.8)	252,313 (12.7)	213,140 (13.8)	162,982 (14.8)	43,712 (16.8)	105,398 (15.7)
Rural	388,011 (17.1)	377,131 (19.0)	309,652 (20.1)	224,569 (20.4)	53,987 (20.8)	139,305 (20.7)
Missings	235,027 (10.4)	182,312 (9.2)	131,461 (8.5)	92,097 (8.4)	20,329 (7.8)	55,356 (8.2)
Smoking status						
Never smoker	612,011 (27.0)	870,939 (43.8)	747,301 (48.4)	542,960 (49.3)	337,067 (50.2)	132,739 (51.1)
Ex‐smoker	106,291 (4.7)	179,021 (9.0)	186,023 (12.1)	160,280 (14.6)	113,721 (16.9)	49,152 (18.9)
Smoker	320,960 (14.1)	336,578 (16.9)	251,177 (16.3)	173,658 (15.8)	103,448 (15.4)	38,201 (14.7)
Missings	1,230,037 (54.2)	603,586 (30.3)	357,935 (23.2)	224,068 (20.4)	117,795 (17.5)	39,457 (15.2)
Alcohol intake risk						
No risk	549,359 (24.2)	807,130 (40.6)	754,580 (48.9)	599,256 (54.4)	549,359 (24.2)	807,130 (40.6)
Low risk	278,465 (12.3)	433,451 (21.8)	398,523 (25.8)	300,346 (27.3)	278,465 (12.3)	433,451 (21.8)
High risk	27,738 (1.2)	45,450 (2.3)	44,691 (2.9)	33,587 (3.1)	27,738 (1.2)	45,450 (2.3)
Missings	1,413,737 (62.3)	704,093 (35.4)	344,642 (22.3)	167,777 (15.2)	1,413,737 (62.3)	704,093 (35.4)
Hormonal therapy (women postmenopausia)						
No consumption	349,825 (95.1)	609,494 (95.5)	552,207 (96.0)	437,679 (96.5)	295,081 (97.0)	132,521 (97.7)
Consumption	18,155 (4.9)	28,650 (4.5)	22,976 (4.0)	15,872 (3.5)	9,080 (3.0)	3,195 (2.4)
Paracetamol						
No consumption	2,204,105 (97.1)	1,738,439 (87.4)	1,244,560 (80.7)	823,167 (74.8)	466,149 (69.4)	161,579 (62.3)
Consumption	65,194 (2.9)	251,685 (12.6)	297,876 (19.3)	277,799 (25.2)	205,882 (30.6)	97,970 (37.7)
Acetylsalicylic acid (ASA)						
No consumption	2,252,990 (99.3)	1,853,313 (93.1)	1,365,347 (88.5)	919,193 (83.5)	524,334 (78.0)	188,274 (72.5)
Consumption	16,309 (0.7)	136,811 (6.9)	177,089 (11.5)	181,773 (16.5)	147,697 (22.0)	71,275 (27.5)
Ibuprofen						
No consumption	2,210,757 (97.4)	1,852,255 (93.1)	1,414,006 (91.7)	1,002,603 (91.1)	612,768 (91.2)	238,670 (92.0)
Consumption	58,542 (2.6)	137,869 (6.9)	128,430 (8.3)	98,363 (8.9)	59,263 (8.8)	20,879 (8.0)
Chronic hepatitis						
No hepatitis	2,244,031 (98.9)	1,956,606 (98.3)	1,512,595 (98.1)	1,078,863 (98.0)	658,974 (98.1)	255,142 (98.3)
Hepatitis B	5249 (0.2)	6773 (0.3)	6281 (0.4)	4898 (0.4)	3261 (0.5)	1303 (0.5)
Hepatitis C	19,703 (0.9)	26,315 (1.3)	23,182 (1.5)	16,928 (1.5)	9626 (1.4)	3043 (1.2)
Other/unspecified hepatitis	316 (0.0)	430 (0.0)	378 (0.0)	277 (0.0)	170 (0.0)	61 (0.0)
Menarche age mean (SD)	12.8 (1.6)	12.8 (1.6)	12.7 (1.6)	12.6 (1.6)	12.6 (1.7)	12.5 (1.7)
Median (IQR)	13 (12–14)	13 (12–14)	13 (12–14)	13 (12–14)	13 (12–14)	12 (11–14)
Missings *n* (%)	776,738 (69.1)	719,289 (70.5)	556,365 (71.3)	412,869 (73.8)	268,926 (75.7)	113,792 (76.3)
Menopause						
No	755,807 (67.3)	381,782 (37.4)	204,691 (26.2)	106.005 (18.9)	51,109 (14.4)	13,515 (9.1)
Yes	367,980 (32.7)	638,144 (62.6)	575,183 (73.8)	453.551 (81.1)	304,161 (85.6)	135,716 (90.9)

*Note*: The data is collected at the beginning of the study or when the participant is diagnosed with 1, 2, 3, 4 or 5 components of MS. *N* total = 3,919,121.

^a^ A participant can contribute in column 0 and in other columns.

Table 2 shows the mean time until cancer diagnosis according to number of components affected. Patients exposed to metabolic syndrome were diagnosed with cancer earlier than those not exposed. Furthermore, the diagnosis was earlier according to number of components added. Specifically, the mean time until cancer diagnosis was (years): 4.35, 4.06, 3.87, 3.68, and 3.46 according to 1, 2, 3, 4, and 5 components respectively.

**TABLE 2:** Mean time until cancer diagnosis according to number of components affected.

	*N*	Age		Cancer risk	Follow‐up until cancer (years)	
		Mean (SD)	Median (IQR)	*N* (%)	Mean (SD)	Median (IQR)
1 component	1,990,124	56.0 (13.9)	55.1 (44.0–64.8)	152,478 (7.66%)	4.35 (2.81)	4.14 (1.91–6.63)
No components	1,990,124	56.0 (13.9)	55.1 (44.0–64.8)	124,777 (6.27%)	4.63 (2.73)	4.49 (2.29–6.84)
2 components	1,542,436	59.6 (13.9)	58.1 (47.9–69.8)	133,821 (8.68%)	4.06 (2.74)	3.76 (1.67–6.20)
No components	1,542,436	59.6 (13.9)	58.1 (47.9–69.8)	113,793 (7.38%)	4.25 (2.69)	4.00 (1.95–6.36)
3 components	1,100,966	61.9 (13.6)	61.1 (50.9–72.1)	100,096 (9.09%)	3.87 (2.68)	3.51 (1.56–5.91)
No components	1,100,966	61.9 (13.6)	61.1 (50.9–72.1)	87,421 (7.94%)	4.03 (2.64)	3.71 (1.78–6.07)
4 components	672,031	63.3 (13.1)	63.0 (53.1–73.2)	60,630 (9.02%)	3.68 (2.61)	3.27 (1.44–5.59)
No components	672,031	63.3 (13.1)	63.0 (53.1–73.2)	52,312 (7.78%)	3.82 (2.58)	3.46 (1.62–5.74)
5 components	259,549	64.2 (12.5)	64.2 (54.8–73.7)	22,112 (8.52%)	3.46 (2.52)	3.04 (1.30–5.28)
No components	259,549	64.2 (12.5)	64.2 (54.8–73.7)	19,023 (7.33%)	3.59 (2.50)	3.15 (1.48–5.35)

*Note*: Participants with altered MS components compared with participants with no components, matched by age and sex. *N*: Number of individuals with MS components (between 1 and 5) and individuals with o MS components matched by age and sex. Age: Mean (SD) and median (IQR) age at which the SM component(s) was diagnosed. Cancer incidence: Number of individuals who have developed cancer and the % of individuals who have developed cancer among the total. Follow‐up until cancer: Mean (standard deviation) and median (IQR) of years of follow‐up of people who have developed cancer.

Abbreviations: IQR, inter quartile range (percentiles 25 and 75); SD, standard deviation.

**FIGURE 1:**
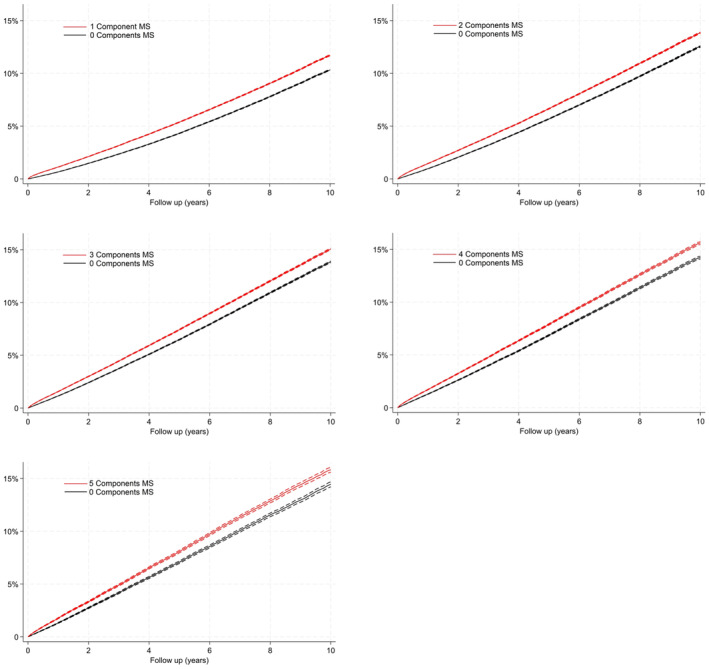
Cumulative incidence function curves for incidence of overall cancer stratified by individuals with MS components, compared to individuals without, matched by age and sex. (The dashed lines represent the 95% CIs).

In Figure 1, we compared the cumulative incidence of any cancer between people with 1, 2, 3, 4, and 5 MS components and people without MS components, matched by age and sex. The cumulative cancer incidence at 5 years in individuals with 1 MS component was 5.4% (vs. 4.3% in participants with no components of MS), and the incidence increased to 11.7% at 10 years (vs. 10.3% in participants with no components MS). These percentages are higher when the individuals have more MS components. For example, in individuals with 5 MS components, the cumulative cancer incidence at 5 years was 8.1% and the cumulative cancer incidence at 10 years was 15.8%. The results are similar when stratifying by cancer type, apart from pre‐menopausal breast cancer (higher incidence in participants with no MS component), pre‐menopausal endometrial cancer (no differences), and prostate cancer (lower incidence in participants with three or more MS components compared to non‐exposed patients) (Figures S1–S17).

**FIGURE 2:**
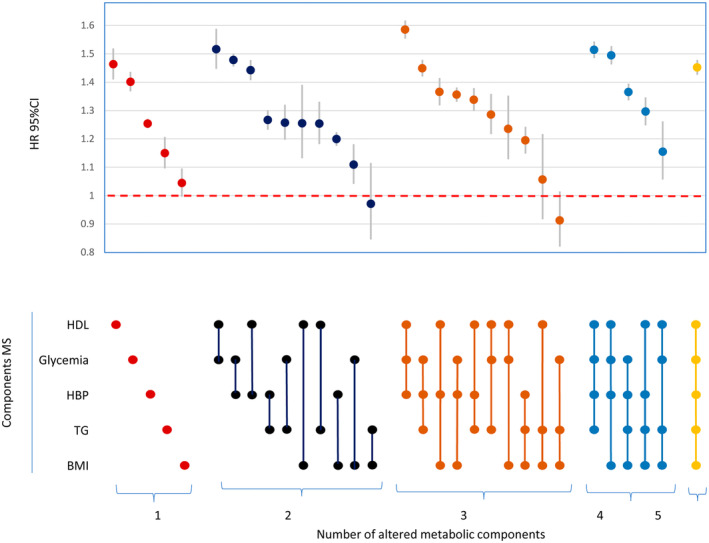
The combined effect of MS components on overall cancer incidence compared with individuals without any MS component. HRs arepresented by circles, with their 95% CIs as vertical lines; HR, hazard ratio; CI, confidence interval. Reference category is 0 components. Cox models adjusted by age, MEDEA Deprivation Index, smoking status and nationality.

We examined the HRs of all possible combinations using a variable with multiple categories, where each category represents a different combination, as can be observed in Figure 2. The group with no MS components was used as the reference category (Figure 2), HDL and Glycemia stood out as individual factors associated with the highest risk of overall cancer (HR 1.46, 95% CI: 1.41–1.52, and HR 1.40, 95% CI: 1.37–1.44 for HDL and Glycemia, respectively). In participants exposed to two components, the combinations with higher HR were HDL+Glycemia (HR 1.52, 95% CI: 1.45–1.59), Glycemia+HBP (HR 1.48, 95% CI: 1.45–1.50), and HDL+HBP (HR 1.44, 95% CI: 1.41–1.48). In participants with three components, the combination with the highest HR was HDL+Glymecia+HBP (HR 1.58, 95% CI: 1.55–1.62). Patterns differed when we analyzed specific cancers, the type of associated component can vary depending on the cancer. (Tables S1–S14). In general, similar results were found for colorectal, prostate, lung cancer, and non‐Hodgkin lymphoma. The HRs in liver, pre and post menopausal endometrial, kidney, bladder, and thyroid cancer were mostly higher than for overall cancer. Similar results were observed in pancreatic cancer in the combinations of 2 components and the same pattern was identified in leukemia, with combinations of 1 component of MS compared with overall cancer. The HRs in the combinations of pre‐ and post‐menopausal breast cancer were mostly lower than the overall cancer. In Hodgkin's lymphoma, only a few statistically significant combinations were detected, with none found in combinations with one component of MS, and three significant HRs identified in two‐component combinations.

**FIGURE 3:**
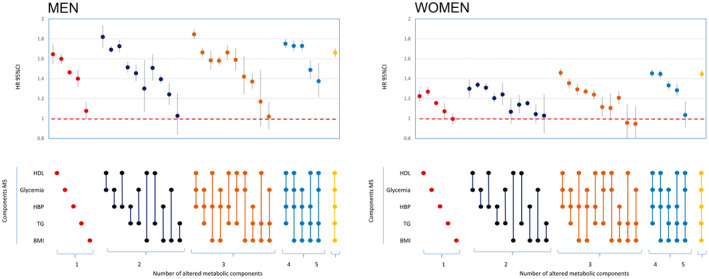
The combined effect of components of MS on overall cancer incidence. Stratified by sex.

Sex‐specific analyses were performed, revealing similar cumulative incidence results for males and females (Figure S18). The total rate of cancer occurrence was greater in males compared to females, and the mean age at cancer diagnosis was higher in females. In the sex‐stratified Cox models depicting all possible MS‐component combinations, the results for women were generally lower than the HRs for men (e.g., HDL+Glycemia combination: women 1.21, men 1.81) (Figure 3). The components in men ordered from the higher HR to the lower were HDL, Glycemia, HBP, TG, and BMI, whereas in women the higher HR to the lower were Glycemia, HDL, HBP, TG, and BMI. In men, the combinations of two components with the highest HRs are: HDL+Glycemia, HDL+HBP, and Glycemia+HBP, while in women, the combinations of two components with the highest HRs are Glycemia+HBP, HDL+HBP, and HDL+Glycemia. In men, the combinations of three components with the highest HRs were HBP+HDL+Glycemia, HBP+Glycemia+TG, and HBP+HDL+TG, while in women, the combinations of three components with the highest HRs were HBP+HDL+Glycemia, HBP+Glycemia+TG, and HBP+Obesity+HDL.

Sensitivity analysis was conducted, excluding cases with non‐melanoma skin cancer (C44). The results from the model incorporating all possible combinations of MS components (Figure 2) were nearly identical when performing the sensitivity analysis (Figure S19). Graphically, no differences were observed, and HRs varied by less than 0.1 points (on average, they varied by 0.04 points) (data not shown). We also repeated the analyses from Figure 2 by creating three age groups: “40–59 years”, “60–79 years”, and “80–99 years” (Figure S20). The results show similar trends regarding the age group. The results also indicate that the HRs are higher in the 80–99 age group and lower in the 40–59 age group. In all three age groups, HDL and Glycemia are the components of MS with the highest HR.

Upon replicating Figure 1 (Cumulative incidence function curves for incidence of overall cancer) and Table 2 (Mean time until cancer diagnosis according to the number of components affected) with the sensitivity analysis, in addition to reducing the sample size, the proportional difference in cancer occurrence between individuals with any MS component and their respective age‐ and sex‐matched individuals with no MS components remained very similar (around 1%). However, in the sensitivity analysis, the cancer risk proportions decreased (data not shown).

## 4 DISCUSSION

In this extensive population‐based study, we found that MS components are correlated with a high incidence of overall cancer and specifically in 10 out of 13 cancers studied, both when presented individually and in combination. These associations occurred independently of certain known factors contributing to cancer risk, like age, alcohol, tobacco, and deprivation status. Our results would also suggest that cancer incidence would increase according to the number of associated MS components.

Among participants with diagnosed cancer, those with more MS components had a shorter time to diagnosis compared to those with fewer or no MS components. However, age appeared to be more influential than the number of affected components on time to diagnosis. A comparison with another study revealed a similar pattern, indicating that individuals with MS have a shorter time between follow‐up initiation and cancer incidence.^26^, ^27^

Consistent with prior research, our findings indicate that MS is associated with an increased risk of different cancer types such as colorectal, liver, pancreatic, endometrial (pre‐ and post‐menopausal), bladder, kidney, thyroid cancers leukemia and non‐Hodgkin lymphoma.^28^, ^29^ The observed association between MS components and cancer incidence was similar to other studies by Li et al.,^16^ where MS was statistically associated with overall cancer in adjusted models, as well as by Mili et al.,^30^ where MS consistently showed a positive correlation with the likelihood of developing common types of cancer. In individuals presenting one MS component, the 5‐year incidence was 5.4% and rising to 8.1% in individuals with five MS components, while in the study of Miyashita et al the 5‐years incidence was 5.0% and 10.3% for one and 5 MS components.^31^ Matched individuals with no components showed slightly lower incidence. Our findings align with other national studies,^29^, ^32^, ^33^ which have revealed a higher 5‐year cancer incidence among those with MS compared to those without it. There are combinations of 3 or 4 components of MS where the risk of cancer is higher than in the combination of 5 components of MS. However, individuals with 5 MS components have, on average, a higher risk than those with four or fewer MS components (see Table S1). This phenomenon also occurs in the study by Oh et al.,^18^ where some combinations of fewer than 5 MS components have a higher risk than the combination of 5 MS components. According to existing literature,^8^ having 3 MS components is considered that the patient has MS, and having 4 or 5 components doesn't significantly increase the risk. Although we followed this premise, we wanted to see if having 4 or 5 components indeed didn't alter the cancer risk, or if, on the contrary, there was an increase in this risk.

In the model with all the combinations of MS components in a single variable with multiple categories, HDL was the MS component with the strongest association with overall cancer risk. This finding in relation to overall cancer is supported by Li et al.^16^ Moreover, various articles have reported different associations between HDL and specific types of cancer, such as endometrial, bladder, pancreatic, kidney, thyroid, colorectal, and lung.^18^, ^27^, ^32^, ^33^, ^34^, ^35^, ^36^ According to our findings, HDL was also associated with these types of cancer.

Glycemia is the second component of MS with a high HR for overall cancer risk. Recent studies specifically investigating the link between glycemia and cancer, have determined that glycemia is linked to a higher likelihood of developing cancer.^37^ These findings are particularly concernig given that the global prevalence of diabetes due to insulin resistance continues to increase.^38^ Other studies have also found that elevated glycemia is linked to a higher risk of specific cancers, such as colorectal, endometrial, bladder, prostate, pancreatic, thyroid, liver, and post‐menopausal breast cancer. These findings are consistent with those of our study.^27^, ^33^, ^39^, ^40^, ^41^, ^42^ Some studies indicate the relationship between glycemia and cancer may be due to insulin resistance that generates hyperinsulinemia, which trigger physiological effects leading to carcinogenesis.^43^

HBP is a common comorbidity in patients suffering from cancer.^30^, ^44^ Our findings are in line with different articles that have indicated a connection between HBP and various types of cancer,^18^, ^33^, ^42^, ^45^, ^46^, ^47^ but the underlying mechanism by which HBP increases the risk for cancer development is unclear.^12^, ^29^

Additionally, elevated TG levels have been linked to a higher risk of certain types of cancer (colorectal, endometrial, prostate, lung, pancreatic cancer, and non‐Hodgkin lymphoma).^33^, ^44^, ^45^, ^47^ In our study TG was associated with colorectal, post‐menopausal endometrial, bladder and prostate cancer.^48^

While waist circumference (WC) is the standard method for determining central adiposity (obesity), we relied on BMI as an indicator of obesity (BMI>30) in accordance with the WHO definition of MS, given that WC data were not accessible for the majority of patients in the SIDIAP database.^49^ In our study, obesity was associated with an increased risk of pre‐menopausal breast, post‐menopausal endometrial and bladder cancer, and inversely associates with lung cancer. This finding is similar to Recalde et al.^10^ who in their study using the SIDIAP database, discovered that increased BMI is positively correlated with the risk of nine cancers (endometrial, kidney, gallbladder, thyroid, colorectal, post‐menopausal breast, multiple myeloma, leukemia, non‐Hodgkin lymphoma) while showing an inverse association with the risk of lung cancer, similar to other population‐based studies.^9^

The combinations of two MS components that included HDL+Glycemia were associated with more cancer types than any other combination. Our study showed that HDL+Glycemia was the combination with the highest positive association with cancer risk. This observation is corroborated by Li et al.,^16^ whose study also revealed that HDL+Glycemia was associated with the highest overall cancer risk. On the other hand, one article reported obesity and glycemia as the combination with the highest cancer risk.^18^ This finding may be attributed to the way in which we defined obesity in this study, given that we used BMI as a proxy to classify obesity and were unable to consider abdominal fat, which is an underlying symptoms of MS. In our study, TG+obesity was the MS component combination associated with only post menopausal endometrial cancer risk. Similarly, Oh TR et al.^18^ found no association with the combination TG‐WC in their adjusted models.

In our study, we observed differences in cancer incidence based on gender, with an overall higher incidence in men compared to women. Additionally, we noted a difference in the mean age at cancer diagnosis, which was higher in women. Our findings align with previous studies that have reported a higher overall cancer incidence in men compared to women.^12^, ^18^, ^29^, ^32^, ^33^, ^39^, ^41^, ^42^ The observed differences in our study highlight the necessity for additional research to elucidate the intricate connection between the components of MS and cancer risk, considering gender‐specific variations and potential modifying factors not fully explored in current literature. The variation between males and females could stem from the interaction of sex hormones^12^, ^33^, ^42^ and fundamental biological and lifestyles differences. In our study, the component most associated with cancer in men is HDL, while in women it is Glycemia. These results are similar to the meta‐analysis of Zhan et al.^50^ in which HDL was the component with the highest risk in men for colorectal cancer and gastrointestinal cancer, meanwhile Glycemia was the component with the highest risk for women in colorectal cancer and gastrointestinal cancer. Futhermore, in the study of Zhan et al., Glycemia was the component with the highest risk for both men and women for pancreatic cancer.

The large sample size of this study, available through SIDIAP, is its main strength. Approximately 75% of the population of Catalonia is included in the SIDIAP database, so the patient data analyzed in this study may be considered representative of the region.^19^ The SIDIAP's cancer diagnoses have been validated and found to be highly consistent with provincial population‐based cancer registries available in Catalonia.^22^ We meticulously analyzed the relationship between the numbers of MS, its individual components, and all the combinations of the MS components, across all cancer types and different types of cancer, going beyond the scope of numerous studies exploring the correlation between MS and cancer. Notably, the level of detail in our analyses surpasses that found in previous research articles on this topic. Our research leveraged a large dataset to assess associations with unparalleled accuracy and power. Another strength of this study is that we analyzed each of the different combinations of MS components for each type of cancer, adding new information, particularly on less frequently studied cancers such as leukemia.

Our study also has limitations. There are individuals without information on some MS components at the beginning of the study. In those cases, we assumed that missing MS component information meant normal levels of that component. This assumption may have generated misclassification bias, as there may be participants who have an altered component that is not recorded in their electronic clinical data. Another limitation is the lack of information on others possible confounding variables, including physical activity, parity, and treatment, which may influence the association between MS and cancer. The consumption of all medications related to MS components was not available, and those that were available could be incomplete. This may also influence the association between MS and cancer. We must also consider the possibility of covariate misclassification bias. For instance, our data on tobacco and alcohol usage exhibit a considerable proportion of missing entries, especially at the beginning of the follow‐up. In those cases, we created a missing category for any missing values. While this approach allowed us to retain as much data as possible for analysis, it introduces some potential bias. Multiple imputation seemed to be a better solution, but it is not feasible due to the complexity of the database, with millions of participants, and where a participant can have multiple entries if their MS condition changes. Using a simpler database, multiple imputation could be performed, but with this simplified database, the necessary analyses for this study could not be conducted.

Additionally, it is worth noting that we classified obesity using BMI instead of abdominal fat (measured as waist circumference) as a criterion for MS. It is crucial to acknowledge that the BMI data obtained from the SIDIAP database exhibit high reliability, and the use of BMI is supported by the WHO. The studies carried out in Spain, which are representative of the population, found that the distribution of BMI is similar to that found in the SIDIAP database.^10^

Some research suggests that the prevalence of MS is on the rise across various regions globally, due in part to the growing prevalence of obesity and other factors that contribute to the development of the condition.^2^, ^3^ This suggests that cancer cases diagnosed every year may be related to MS. To reduce the occurrence of MS, public health initiatives may prioritize encouraging healthy lifestyle choices, including engaging exercise, adopting a balanced diet, and abstaining from tobacco consumption. Public health campaigns can also educate people about the risks of MS and provide them with information about how to prevent or manage the condition. Additionally, public health programs can target specific groups of people prone to MS onset, such as individuals with obesity or who have a family history of the condition. Recognizing the significance of MS as a contributing factor to certain types of cancer is vital for early detection and treatment of cancer.

In conclusion, the presence of one or more MS components increases the incidence of acquiring a minimum of 10 types of cancer, and these incidences vary according to the type of component included. Some sex differences are also observed. Our findings suggest that implemention of preventive measures targeting specific MS components could be an initiative that would help to reduce the incidence of cancer.

## AUTHOR CONTRIBUTIONS

**Tomàs López‐Jiménez:** Conceptualization (equal); data curation (lead); formal analysis (equal); investigation (equal); methodology (equal); software (lead); supervision (equal); validation (equal); visualization (equal); writing – original draft (equal); writing – review and editing (equal). **Oleguer Plana‐Ripoll:** Conceptualization (equal); formal analysis (equal); methodology (equal); software (equal); writing – review and editing (equal). **Talita Duarte‐Salles:** Conceptualization (equal); writing – review and editing (equal). **Martina Recalde:** Conceptualization (equal); writing – review and editing (equal). **Matthew Bennett:** Writing – review and editing (equal). **Francesc Xavier‐Cos:** Conceptualization (equal); writing – review and editing (equal). **Diana Puente:** Conceptualization (equal); funding acquisition (lead); methodology (equal); resources (lead); writing – original draft (equal); writing – review and editing (equal).

## FUNDING INFORMATION

The project was funded from the Carlos III Institute of Health, (Spain) (reference PI17/00914). MR work was supported by the Wereld Kanker Onderzoek Fonds (WKOF), through the World Cancer Research Fund International grant program [grant number: 2017/1630].

## CONFLICT OF INTEREST STATEMENT

The authors have no conflict of interest to declare.

## ETHICS STATEMENT

This study adheres to both national and international guidelines, including the Declaration of Helsinki and Principles of Good Research Practice. In compliance with European and Spanish laws regarding confidentiality and data protection ([EU] 2016/679), all data within SIDIAP is consistently pseudo‐anonymized. Hence, obtaining consent from the participants was deemed unnecessary and was waived by the Clinical Ethics Committee at IDIAPJGol. To establish a connection with the CMBD database, SIDIAP utilizes a third‐party service to maintain confidentiality. Approval for the study protocol was granted by the Clinical Research Ethics Committee of IDIAPJGol (P17/212) on November 29, 2017. Anonymity and confidentiality of data and medical records were ensured throughout, following the guidelines outlined in the Organic Law 15/1999 on the Protection of Personal Data (http://www.boe.es/boe/dias/1999/12/14/pdfs/A43088‐43099.pdf).

## Supporting information


Data S1:

## Data Availability

The data utilized for analysis in this study were sourced from electronic records within the primary healthcare clinical history. Limitations affect the accessibility of this data, rendering it unavailable to the public. Nevertheless, authors may obtain the data through a reasonable request.
