# Supplementary material for: Exploring the association between metabolic syndrome, its components and subsequent cancer incidence: A cohort study in Catalonia
Source: Cancer Med. 2024 Aug 16;13(16):e7400. doi: 10.1002/cam4.7400 (PMC11327772; doi:10.1002/cam4.7400)
Supplement: Supplementary file 1 — Data S1: [file CAM4-13-e7400-s001.docx]

**Supplementary Table 1**. Colorectal cancer. The combined effect of components of MS on colorectal cancer incidence

|  | **Cancer risk** | | | |
| --- | --- | --- | --- | --- |
| **Metabolic syndrome** | **HR** | **95%** | **CI** | **Pvalue** |
| 0 components | 1.00 |  |  |  |
| 1 component | 1.24 | 1.20 | 1.29 | <0.001 |
| HDL | 1.44 | 1.27 | 1.63 | <0.001 |
| Glycemia | 1.57 | 1.46 | 1.69 | <0.001 |
| HBP | 1.21 | 1.16 | 1.26 | <0.001 |
| TG | 1.16 | 1.00 | 1.35 | 0.047 |
| Obesity | 0.99 | 0.84 | 1.15 | 0.871 |
| 2 components | 1.39 | 1.33 | 1.45 | <0.001 |
| HDL+Glycemia | 1.65 | 1.44 | 1.90 | <0.001 |
| HBP+Glycemia | 1.54 | 1.47 | 1.61 | <0.001 |
| HBP+HDL | 1.32 | 1.22 | 1.42 | <0.001 |
| HBP+TG | 1.16 | 1.06 | 1.26 | 0.001 |
| Glycemia+TG | 1.27 | 1.10 | 1.47 | 0.001 |
| Obesity+HDL | 1.32 | 0.94 | 1.85 | 0.110 |
| HDL+TG | 1.23 | 1.01 | 1.49 | 0.035 |
| HBP+ Obesity | 1.20 | 1.13 | 1.28 | <0.001 |
| Obesity +Glycemia | 1.33 | 1.12 | 1.58 | 0.001 |
| Obesity +TG | 1.02 | 0.67 | 1.56 | 0.914 |
| 3 components | 1.51 | 1.44 | 1.57 | <0.001 |
| HBP+HDL+Glycemia | 1.67 | 1.58 | 1.78 | <0.001 |
| HBP+Glycemia+TG | 1.55 | 1.46 | 1.64 | <0.001 |
| HBP+ Obesity +HDL | 1.47 | 1.32 | 1.63 | <0.001 |
| HBP+ Obesity +Glycemia | 1.55 | 1.47 | 1.64 | <0.001 |
| HBP+HDL+TG | 1.19 | 1.08 | 1.31 | <0.001 |
| HDL+Glycemia+TG | 1.27 | 1.07 | 1.50 | 0.006 |
| Obesity +HDL+Glycemia | 1.75 | 1.37 | 2.22 | <0.001 |
| HBP+ Obesity +TG | 1.16 | 1.03 | 1.31 | 0.015 |
| Obesity +HDL+TG | 1.28 | 0.84 | 1.94 | 0.252 |
| Obesity +Glycemia+TG | 1.05 | 0.78 | 1.41 | 0.748 |
| 4 components | 1.57 | 1.50 | 1.64 | <0.001 |
| HBP+HDL+Glycemia+TG | 1.57 | 1.49 | 1.66 | <0.001 |
| HBP+ Obesity +HDL+Glycemia | 1.72 | 1.62 | 1.83 | <0.001 |
| HBP+ Obesity +Glycemia+TG | 1.54 | 1.45 | 1.64 | <0.001 |
| HBP+ Obesity +HDL+TG | 1.21 | 1.07 | 1.36 | 0.002 |
| Obesity +HDL+Glycemia+TG | 1.37 | 1.07 | 1.77 | 0.013 |
| 5 components | 1.61 | 1.53 | 1.70 | <0.001 |

Model adjusted by age, sex, MEDEA Deprivation Index, smoking status, alcohol consumption and nationality

**Supplementary Table 2.** Liver cancer. The combined effect of components of MS on liver cancer incidence

|  | **Cancer risk** | | | |
| --- | --- | --- | --- | --- |
| **Metabolic syndrome** | **HR** | **95%** | **CI** | **Pvalue** |
| 0 components | 1.00 |  |  |  |
| 1 component | 2.42 | 2.16 | 2.71 | <0.001 |
| HDL | 2.14 | 1.52 | 3.01 | <0.001 |
| Glycemia | 2.32 | 1.89 | 2.84 | <0.001 |
| HBP | 2.64 | 2.35 | 2.96 | <0.001 |
| TG | 0.79 | 0.43 | 1.43 | 0.433 |
| Obesity | 0.90 | 0.50 | 1.59 | 0.708 |
| 2 components | 3.21 | 2.86 | 3.61 | <0.001 |
| HDL+Glycemia | 3.50 | 2.53 | 4.84 | <0.001 |
| HBP+Glycemia | 3.84 | 3.38 | 4.36 | <0.001 |
| HBP+HDL | 3.79 | 3.16 | 4.56 | <0.001 |
| HBP+TG | 1.70 | 1.32 | 2.20 | <0.001 |
| Glycemia+TG | 2.33 | 1.61 | 3.38 | <0.001 |
| Obesity+HDL | 0.46 | 0.07 | 3.30 | 0.442 |
| HDL+TG | 1.30 | 0.69 | 2.43 | 0.416 |
| HBP+ Obesity | 3.24 | 2.78 | 3.79 | <0.001 |
| Obesity +Glycemia | 1.08 | 0.54 | 2.18 | 0.827 |
| Obesity +TG | 0.59 | 0.08 | 4.21 | 0.600 |
| 3 components | 3.64 | 3.23 | 4.09 | <0.001 |
| HBP+HDL+Glycemia | 6.18 | 5.37 | 7.12 | <0.001 |
| HBP+Glycemia+TG | 3.03 | 2.57 | 3.58 | <0.001 |
| HBP+ Obesity +HDL | 3.31 | 2.52 | 4.35 | <0.001 |
| HBP+ Obesity +Glycemia | 3.96 | 3.43 | 4.57 | <0.001 |
| HBP+HDL+TG | 1.80 | 1.35 | 2.41 | <0.001 |
| HDL+Glycemia+TG | 1.82 | 1.12 | 2.96 | 0.015 |
| Obesity +HDL+Glycemia | 1.26 | 0.47 | 3.37 | 0.647 |
| HBP+ Obesity +TG | 1.92 | 1.34 | 2.74 | <0.001 |
| Obesity +HDL+TG | --- | --- | --- | --- |
| Obesity +Glycemia+TG | 1.54 | 0.64 | 3.73 | 0.337 |
| 4 components | 3.39 | 2.99 | 3.85 | <0.001 |
| HBP+HDL+Glycemia+TG | 3.64 | 3.12 | 4.23 | <0.001 |
| HBP+ Obesity +HDL+Glycemia | 4.61 | 3.94 | 5.40 | <0.001 |
| HBP+ Obesity +Glycemia+TG | 2.88 | 2.42 | 3.43 | <0.001 |
| HBP+ Obesity +HDL+TG | 1.85 | 1.28 | 2.67 | 0.001 |
| Obesity +HDL+Glycemia+TG | 1.98 | 0.94 | 4.19 | 0.073 |
| 5 components | 3.72 | 3.23 | 4.30 | <0.001 |

Model adjusted by age, sex, MEDEA Deprivation Index, smoking status, alcohol consumption, nationality, hepatitis and other liver disease

**Supplementary Table 3.** Pancreas cancer. The combined effect of components of MS on pancreas cancer incidence

|  | **Cancer risk** | | | |
| --- | --- | --- | --- | --- |
| **Metabolic syndrome** | **HR** | **95%** | **CI** | **Pvalue** |
| 0 components | 1.00 |  |  |  |
| 1 component | 1.80 | 1.61 | 2.03 | <0.001 |
| HDL | 1.71 | 1.18 | 2.46 | 0.004 |
| Glycemia | 2.80 | 2.30 | 3.40 | <0.001 |
| HBP | 1.80 | 1.59 | 2.04 | <0.001 |
| TG | 1.01 | 0.58 | 1.76 | 0.960 |
| Obesity | 0.96 | 0.55 | 1.67 | 0.896 |
| 2 components | 2.98 | 2.65 | 3.35 | <0.001 |
| HDL+Glycemia | 3.76 | 2.72 | 5.20 | <0.001 |
| HBP+Glycemia | 4.25 | 3.74 | 4.83 | <0.001 |
| HBP+HDL | 2.09 | 1.68 | 2.61 | <0.001 |
| HBP+TG | 1.88 | 1.46 | 2.41 | <0.001 |
| Glycemia+TG | 2.22 | 1.47 | 3.35 | <0.001 |
| Obesity+HDL | 0.85 | 0.21 | 3.40 | 0.814 |
| HDL+TG | 1.31 | 0.70 | 2.44 | 0.405 |
| HBP+ Obesity | 2.09 | 1.75 | 2.49 | <0.001 |
| Obesity +Glycemia | 0.74 | 0.31 | 1.79 | 0.503 |
| Obesity +TG | 0.63 | 0.09 | 4.50 | 0.647 |
| 3 components | 3.43 | 3.04 | 3.87 | <0.001 |
| HBP+HDL+Glycemia | 4.68 | 4.02 | 5.45 | <0.001 |
| HBP+Glycemia+TG | 4.41 | 3.78 | 5.14 | <0.001 |
| HBP+ Obesity +HDL | 1.63 | 1.14 | 2.33 | 0.007 |
| HBP+ Obesity +Glycemia | 3.57 | 3.07 | 4.14 | <0.001 |
| HBP+HDL+TG | 1.92 | 1.45 | 2.52 | <0.001 |
| HDL+Glycemia+TG | 3.04 | 2.04 | 4.51 | <0.001 |
| Obesity +HDL+Glycemia | 1.68 | 0.70 | 4.07 | 0.248 |
| HBP+ Obesity +TG | 1.73 | 1.19 | 2.51 | 0.004 |
| Obesity +HDL+TG | 0.69 | 0.10 | 4.94 | 0.716 |
| Obesity +Glycemia+TG | 1.82 | 0.75 | 4.40 | 0.184 |
| 4 components | 3.95 | 3.48 | 4.48 | <0.001 |
| HBP+HDL+Glycemia+TG | 4.97 | 4.30 | 5.73 | <0.001 |
| HBP+ Obesity +HDL+Glycemia | 4.12 | 3.50 | 4.85 | <0.001 |
| HBP+ Obesity +Glycemia+TG | 3.59 | 3.04 | 4.25 | <0.001 |
| HBP+ Obesity +HDL+TG | 2.02 | 1.44 | 2.84 | <0.001 |
| Obesity +HDL+Glycemia+TG | 2.12 | 1.00 | 4.47 | 0.049 |
| 5 components | 4.35 | 3.79 | 5.00 | <0.001 |

Model adjusted by age, sex, MEDEA Deprivation Index, smoking status, alcohol consumption and nationality

**Supplementary Table 4.** Breast pre-menopause cancer. The combined effect of components of MS on breast pre-menopause cancer incidence

|  | **Cancer risk** | | | |
| --- | --- | --- | --- | --- |
| **Metabolic syndrome** | **HR** | **95%** | **CI** | **Pvalue** |
| 0 components | 1.00 |  |  |  |
| 1 component | 0.93 | 0.88 | 0.99 | 0.014 |
| HDL | 0.94 | 0.84 | 1.05 | 0.266 |
| Glycemia | 1.04 | 0.91 | 1.18 | 0.597 |
| HBP | 0.95 | 0.89 | 1.01 | 0.102 |
| TG | 0.89 | 0.70 | 1.13 | 0.346 |
| Obesity | 0.71 | 0.60 | 0.85 | <0.001 |
| 2 components | 0.80 | 0.74 | 0.86 | <0.001 |
| HDL+Glycemia | 0.93 | 0.74 | 1.18 | 0.555 |
| HBP+Glycemia | 0.79 | 0.67 | 0.94 | 0.008 |
| HBP+HDL | 0.92 | 0.80 | 1.06 | 0.232 |
| HBP+TG | 0.91 | 0.70 | 1.18 | 0.476 |
| Glycemia+TG | 0.69 | 0.40 | 1.18 | 0.176 |
| Obesity+HDL | 0.58 | 0.41 | 0.81 | 0.002 |
| HDL+TG | 0.59 | 0.43 | 0.80 | 0.001 |
| HBP+ Obesity | 0.74 | 0.64 | 0.85 | <0.001 |
| Obesity +Glycemia | 0.71 | 0.49 | 1.03 | 0.071 |
| Obesity +TG | 1.28 | 0.77 | 2.13 | 0.340 |
| 3 components | 0.63 | 0.56 | 0.71 | <0.001 |
| HBP+HDL+Glycemia | 0.70 | 0.54 | 0.91 | 0.008 |
| HBP+Glycemia+TG | 0.71 | 0.44 | 1.15 | 0.163 |
| HBP+ Obesity +HDL | 0.53 | 0.41 | 0.68 | <0.001 |
| HBP+ Obesity +Glycemia | 0.62 | 0.47 | 0.81 | <0.001 |
| HBP+HDL+TG | 0.61 | 0.45 | 0.83 | 0.002 |
| HDL+Glycemia+TG | 0.73 | 0.49 | 1.10 | 0.136 |
| Obesity +HDL+Glycemia | 0.46 | 0.28 | 0.77 | 0.003 |
| HBP+ Obesity +TG | 0.88 | 0.59 | 1.31 | 0.522 |
| Obesity +HDL+TG | 0.76 | 0.47 | 1.25 | 0.285 |
| Obesity +Glycemia+TG | 0.30 | 0.07 | 1.19 | 0.086 |
| 4 components | 0.71 | 0.61 | 0.82 | <0.001 |
| HBP+HDL+Glycemia+TG | 0.70 | 0.55 | 0.89 | 0.003 |
| HBP+ Obesity +HDL+Glycemia | 0.77 | 0.58 | 1.04 | 0.089 |
| HBP+ Obesity +Glycemia+TG | 0.85 | 0.54 | 1.31 | 0.454 |
| HBP+ Obesity +HDL+TG | 0.72 | 0.52 | 1.00 | 0.048 |
| Obesity +HDL+Glycemia+TG | 0.37 | 0.18 | 0.73 | 0.004 |
| 5 components | 0.69 | 0.52 | 0.90 | 0.006 |

Model adjusted by age, sex, MEDEA Deprivation Index, smoking status, alcohol consumption and nationality

**Supplementary Table 5.** Breast post-menopause cancer. The combined effect of components of MS on breast post-menopause cancer incidence

|  | **Cancer risk** | | | |
| --- | --- | --- | --- | --- |
| **Metabolic syndrome** | **HR** | **95%** | **CI** | **Pvalue** |
| 0 components | 1.00 |  |  |  |
| 1 component | 1.07 | 1.03 | 1.11 | <0.001 |
| HDL | 0.90 | 0.80 | 1.01 | 0.078 |
| Glycemia | 1.10 | 1.02 | 1.19 | 0.015 |
| HBP | 1.09 | 1.05 | 1.13 | <0.001 |
| TG | 0.92 | 0.78 | 1.08 | 0.288 |
| Obesity | 1.08 | 0.96 | 1.22 | 0.186 |
| 2 components | 1.03 | 0.99 | 1.08 | 0.098 |
| HDL+Glycemia | 0.94 | 0.80 | 1.11 | 0.462 |
| HBP+Glycemia | 1.07 | 1.01 | 1.13 | 0.012 |
| HBP+HDL | 0.94 | 0.86 | 1.01 | 0.098 |
| HBP+TG | 1.03 | 0.94 | 1.13 | 0.477 |
| Glycemia+TG | 1.23 | 1.04 | 1.46 | 0.018 |
| Obesity+HDL | 1.01 | 0.77 | 1.34 | 0.937 |
| HDL+TG | 0.82 | 0.67 | 0.99 | 0.036 |
| HBP+ Obesity | 1.09 | 1.03 | 1.15 | 0.004 |
| Obesity +Glycemia | 0.94 | 0.79 | 1.14 | 0.541 |
| Obesity +TG | 0.82 | 0.54 | 1.25 | 0.356 |
| 3 components | 1.09 | 1.04 | 1.13 | <0.001 |
| HBP+HDL+Glycemia | 1.12 | 1.04 | 1.20 | 0.002 |
| HBP+Glycemia+TG | 1.14 | 1.05 | 1.23 | 0.001 |
| HBP+ Obesity +HDL | 1.09 | 0.98 | 1.20 | 0.110 |
| HBP+ Obesity +Glycemia | 1.13 | 1.07 | 1.20 | <0.001 |
| HBP+HDL+TG | 0.99 | 0.90 | 1.09 | 0.787 |
| HDL+Glycemia+TG | 0.95 | 0.79 | 1.14 | 0.585 |
| Obesity +HDL+Glycemia | 0.91 | 0.69 | 1.20 | 0.514 |
| HBP+ Obesity +TG | 1.04 | 0.92 | 1.17 | 0.543 |
| Obesity +HDL+TG | 1.00 | 0.70 | 1.43 | 0.999 |
| Obesity +Glycemia+TG | 0.89 | 0.64 | 1.25 | 0.511 |
| 4 components | 1.10 | 1.05 | 1.16 | <0.001 |
| HBP+HDL+Glycemia+TG | 1.07 | 1.00 | 1.15 | 0.042 |
| HBP+ Obesity +HDL+Glycemia | 1.19 | 1.11 | 1.27 | <0.001 |
| HBP+ Obesity +Glycemia+TG | 1.16 | 1.08 | 1.25 | <0.001 |
| HBP+ Obesity +HDL+TG | 0.96 | 0.85 | 1.08 | 0.472 |
| Obesity +HDL+Glycemia+TG | 0.86 | 0.65 | 1.12 | 0.254 |
| 5 components | 1.14 | 1.08 | 1.20 | <0.001 |

Model adjusted by age, sex, MEDEA Deprivation Index, smoking status, alcohol consumption and nationality

**Supplementary Table 6**. Endometrial pre-menopause cancer. The combined effect of components of MS on endometrial pre-menopause cancer incidence

|  | **Cancer risk** | | | |
| --- | --- | --- | --- | --- |
| **Metabolic syndrome** | **HR** | **95%** | **CI** | **Pvalue** |
| 0 components | 1.00 |  |  |  |
| 1 component | 1.38 | 1.09 | 1.74 | 0.007 |
| HDL | 1.67 | 1.09 | 2.57 | 0.018 |
| Glycemia | 2.12 | 1.36 | 3.31 | 0.001 |
| HBP | 1.20 | 0.90 | 1.60 | 0.223 |
| TG | 0.94 | 0.3 | 2.94 | 0.912 |
| Obesity | 1.29 | 0.68 | 2.44 | 0.440 |
| 2 components | 1.38 | 1.01 | 1.87 | 0.041 |
| HDL+Glycemia | 0.29 | 0.04 | 2.11 | 0.223 |
| HBP+Glycemia | 1.49 | 0.81 | 2.76 | 0.203 |
| HBP+HDL | 1.19 | 0.65 | 2.21 | 0.574 |
| HBP+TG | 1.16 | 0.37 | 3.63 | 0.804 |
| Glycemia+TG | 1.17 | 0.16 | 8.34 | 0.878 |
| Obesity+HDL | 1.69 | 0.63 | 4.57 | 0.300 |
| HDL+TG | 1.70 | 0.70 | 4.13 | 0.244 |
| HBP+ Obesity | 1.78 | 1.11 | 2.84 | 0.016 |
| Obesity +Glycemia | 1.15 | 0.28 | 4.62 | 0.849 |
| Obesity +TG | --- | --- | --- | --- |
| 3 components | 2.23 | 1.59 | 3.11 | <0.001 |
| HBP+HDL+Glycemia | 2.27 | 1.11 | 4.64 | 0.024 |
| HBP+Glycemia+TG | 0.96 | 0.13 | 6.88 | 0.969 |
| HBP+ Obesity +HDL | 1.46 | 0.68 | 3.13 | 0.330 |
| HBP+ Obesity +Glycemia | 3.46 | 1.95 | 6.13 | <0.001 |
| HBP+HDL+TG | 1.74 | 0.71 | 4.26 | 0.222 |
| HDL+Glycemia+TG | 2.12 | 0.67 | 6.64 | 0.199 |
| Obesity +HDL+Glycemia | 3.52 | 1.44 | 8.59 | 0.006 |
| HBP+ Obesity +TG | 0.88 | 0.12 | 6.33 | 0.902 |
| Obesity +HDL+TG | 2.24 | 0.56 | 9.07 | 0.257 |
| Obesity +Glycemia+TG | 6.61 | 1.63 | 26.7 | 0.008 |
| 4 components | 2.11 | 1.35 | 3.31 | 0.001 |
| HBP+HDL+Glycemia+TG | 0.91 | 0.22 | 3.68 | 0.894 |
| HBP+ Obesity +HDL+Glycemia | 3.23 | 1.85 | 5.64 | <0.001 |
| HBP+ Obesity +Glycemia+TG | 0.97 | 0.14 | 6.96 | 0.977 |
| HBP+ Obesity +HDL+TG | 1.26 | 0.40 | 3.97 | 0.692 |
| Obesity +HDL+Glycemia+TG | 3.07 | 0.98 | 9.67 | 0.055 |
| 5 components | 3.77 | 2.11 | 6.72 | <0.001 |

Model adjusted by age, sex, MEDEA Deprivation Index, smoking status, alcohol consumption and nationality

**Supplementary Table 7.** Endometrial post-menopause cancer. The combined effect of components of MS on endometrial post-menopause cancer incidence

|  | **Cancer risk** | | | |
| --- | --- | --- | --- | --- |
| **Metabolic syndrome** | **HR** | **95%** | **CI** | **Pvalue** |
| 0 components | 1.00 |  |  |  |
| 1 component | 1.46 | 1.30 | 1.63 | <0.001 |
| HDL | 1.86 | 1.40 | 2.49 | <0.001 |
| Glycemia | 1.67 | 1.34 | 2.07 | <0.001 |
| HBP | 1.37 | 1.21 | 1.54 | <0.001 |
| TG | 2.36 | 1.68 | 3.33 | <0.001 |
| Obesity | 1.89 | 1.39 | 2.58 | <0.001 |
| 2 components | 1.98 | 1.76 | 2.22 | <0.001 |
| HDL+Glycemia | 2.71 | 1.95 | 3.77 | <0.001 |
| HBP+Glycemia | 1.94 | 1.68 | 2.23 | <0.001 |
| HBP+HDL | 1.68 | 1.36 | 2.08 | <0.001 |
| HBP+TG | 1.55 | 1.20 | 2.01 | 0.001 |
| Glycemia+TG | 1.52 | 0.91 | 2.54 | 0.112 |
| Obesity+HDL | 1.70 | 0.80 | 3.58 | 0.165 |
| HDL+TG | 0.88 | 0.47 | 1.65 | 0.697 |
| HBP+ Obesity | 2.38 | 2.06 | 2.76 | <0.001 |
| Obesity +Glycemia | 1.89 | 1.22 | 2.92 | 0.005 |
| Obesity +TG | 2.54 | 1.13 | 5.68 | 0.024 |
| 3 components | 2.48 | 2.20 | 2.79 | <0.001 |
| HBP+HDL+Glycemia | 2.26 | 1.88 | 2.71 | <0.001 |
| HBP+Glycemia+TG | 1.75 | 1.41 | 2.18 | <0.001 |
| HBP+ Obesity +HDL | 2.77 | 2.20 | 3.49 | <0.001 |
| HBP+ Obesity +Glycemia | 3.25 | 2.83 | 3.74 | <0.001 |
| HBP+HDL+TG | 1.68 | 1.30 | 2.17 | <0.001 |
| HDL+Glycemia+TG | 1.68 | 1.06 | 2.66 | 0.026 |
| Obesity +HDL+Glycemia | 3.43 | 2.11 | 5.56 | <0.001 |
| HBP+ Obesity +TG | 2.15 | 1.60 | 2.88 | <0.001 |
| Obesity +HDL+TG | 1.18 | 0.38 | 3.67 | 0.776 |
| Obesity +Glycemia+TG | 2.02 | 0.96 | 4.26 | 0.065 |
| 4 components | 3.10 | 2.75 | 3.50 | <0.001 |
| HBP+HDL+Glycemia+TG | 2.14 | 1.80 | 2.54 | <0.001 |
| HBP+ Obesity +HDL+Glycemia | 4.42 | 3.83 | 5.10 | <0.001 |
| HBP+ Obesity +Glycemia+TG | 2.96 | 2.50 | 3.52 | <0.001 |
| HBP+ Obesity +HDL+TG | 2.77 | 2.17 | 3.53 | <0.001 |
| Obesity +HDL+Glycemia+TG | 2.53 | 1.48 | 4.30 | 0.001 |
| 5 components | 4.15 | 3.65 | 4.73 | <0.001 |

Model adjusted by age, sex, MEDEA Deprivation Index, smoking status, alcohol consumption and nationality

**Supplementary Table 8.** Bladder cancer. The combined effect of components of MS on bladder cancer incidence

|  | **Cancer risk** | | | |
| --- | --- | --- | --- | --- |
| **Metabolic syndrome** | **HR** | **95%** | **CI** | **Pvalue** |
| 0 components | 1.00 |  |  |  |
| 1 component | 1.44 | 1.36 | 1.52 | <0.001 |
| HDL | 1.69 | 1.42 | 2.02 | <0.001 |
| Glycemia | 1.55 | 1.41 | 1.71 | <0.001 |
| HBP | 1.42 | 1.34 | 1.50 | <0.001 |
| TG | 1.33 | 1.09 | 1.61 | 0.004 |
| Obesity | 1.27 | 1.03 | 1.56 | 0.027 |
| 2 components | 1.59 | 1.50 | 1.68 | <0.001 |
| HDL+Glycemia | 1.95 | 1.63 | 2.33 | <0.001 |
| HBP+Glycemia | 1.70 | 1.59 | 1.80 | <0.001 |
| HBP+HDL | 1.49 | 1.34 | 1.66 | <0.001 |
| HBP+TG | 1.47 | 1.32 | 1.64 | <0.001 |
| Glycemia+TG | 1.59 | 1.34 | 1.88 | <0.001 |
| Obesity+HDL | 1.51 | 0.91 | 2.50 | 0.115 |
| HDL+TG | 1.81 | 1.43 | 2.28 | <0.001 |
| HBP+ Obesity | 1.33 | 1.22 | 1.45 | <0.001 |
| Obesity +Glycemia | 1.17 | 0.90 | 1.52 | 0.234 |
| Obesity +TG | 1.13 | 0.64 | 1.99 | 0.675 |
| 3 components | 1.70 | 1.60 | 1.80 | <0.001 |
| HBP+HDL+Glycemia | 1.89 | 1.74 | 2.04 | <0.001 |
| HBP+Glycemia+TG | 1.76 | 1.63 | 1.90 | <0.001 |
| HBP+ Obesity +HDL | 1.68 | 1.44 | 1.95 | <0.001 |
| HBP+ Obesity +Glycemia | 1.51 | 1.41 | 1.63 | <0.001 |
| HBP+HDL+TG | 1.73 | 1.54 | 1.95 | <0.001 |
| HDL+Glycemia+TG | 2.10 | 1.75 | 2.51 | <0.001 |
| Obesity +HDL+Glycemia | 1.60 | 1.11 | 2.29 | 0.011 |
| HBP+ Obesity +TG | 1.50 | 1.29 | 1.75 | <0.001 |
| Obesity +HDL+TG | 1.22 | 0.63 | 2.34 | 0.557 |
| Obesity +Glycemia+TG | 1.53 | 1.10 | 2.12 | 0.011 |
| 4 components | 1.70 | 1.59 | 1.80 | <0.001 |
| HBP+HDL+Glycemia+TG | 1.81 | 1.68 | 1.96 | <0.001 |
| HBP+ Obesity +HDL+Glycemia | 1.66 | 1.52 | 1.81 | <0.001 |
| HBP+ Obesity +Glycemia+TG | 1.59 | 1.46 | 1.72 | <0.001 |
| HBP+ Obesity +HDL+TG | 1.53 | 1.30 | 1.80 | <0.001 |
| Obesity +HDL+Glycemia+TG | 1.87 | 1.37 | 2.54 | <0.001 |
| 5 components | 1.71 | 1.59 | 1.84 | <0.001 |

Model adjusted by age, sex, MEDEA Deprivation Index, smoking status, alcohol consumption and nationality

**Supplementary Table 9.** Kidney cancer. The combined effect of components of MS on kidney cancer incidence

|  | **Cancer risk** | | | |
| --- | --- | --- | --- | --- |
| **Metabolic syndrome** | **HR** | **95%** | **CI** | **Pvalue** |
| 0 components | 1.00 |  |  |  |
| 1 component | 1.76 | 1.61 | 1.92 | <0.001 |
| HDL | 1.72 | 1.32 | 2.23 | <0.001 |
| Glycemia | 1.42 | 1.18 | 1.70 | <0.001 |
| HBP | 1.85 | 1.68 | 2.03 | <0.001 |
| TG | 1.33 | 0.95 | 1.85 | 0.094 |
| Obesity | 1.27 | 0.91 | 1.78 | 0.156 |
| 2 components | 1.87 | 1.70 | 2.06 | <0.001 |
| HDL+Glycemia | 2.22 | 1.65 | 2.98 | <0.001 |
| HBP+Glycemia | 1.87 | 1.67 | 2.09 | <0.001 |
| HBP+HDL | 2.65 | 2.26 | 3.10 | <0.001 |
| HBP+TG | 1.59 | 1.31 | 1.94 | <0.001 |
| Glycemia+TG | 1.27 | 0.88 | 1.83 | 0.204 |
| Obesity+HDL | 1.02 | 0.42 | 2.46 | 0.961 |
| HDL+TG | 2.08 | 1.47 | 2.95 | <0.001 |
| HBP+ Obesity | 1.77 | 1.54 | 2.04 | <0.001 |
| Obesity +Glycemia | 1.03 | 0.62 | 1.72 | 0.900 |
| Obesity +TG | 1.41 | 0.58 | 3.39 | 0.447 |
| 3 components | 2.13 | 1.93 | 2.36 | <0.001 |
| HBP+HDL+Glycemia | 2.39 | 2.08 | 2.76 | <0.001 |
| HBP+Glycemia+TG | 1.86 | 1.60 | 2.16 | <0.001 |
| HBP+ Obesity +HDL | 2.84 | 2.30 | 3.52 | <0.001 |
| HBP+ Obesity +Glycemia | 2.08 | 1.82 | 2.37 | <0.001 |
| HBP+HDL+TG | 2.21 | 1.81 | 2.69 | <0.001 |
| HDL+Glycemia+TG | 2.05 | 1.47 | 2.87 | <0.001 |
| Obesity +HDL+Glycemia | 2.19 | 1.29 | 3.71 | 0.004 |
| HBP+ Obesity +TG | 2.15 | 1.68 | 2.76 | <0.001 |
| Obesity +HDL+TG | 0.98 | 0.31 | 3.04 | 0.969 |
| Obesity +Glycemia+TG | 1.43 | 0.74 | 2.77 | 0.284 |
| 4 components | 2.51 | 2.26 | 2.79 | <0.001 |
| HBP+HDL+Glycemia+TG | 2.39 | 2.09 | 2.73 | <0.001 |
| HBP+ Obesity +HDL+Glycemia | 3.06 | 2.67 | 3.51 | <0.001 |
| HBP+ Obesity +Glycemia+TG | 2.15 | 1.85 | 2.49 | <0.001 |
| HBP+ Obesity +HDL+TG | 2.88 | 2.31 | 3.59 | <0.001 |
| Obesity +HDL+Glycemia+TG | 1.98 | 1.16 | 3.36 | 0.012 |
| 5 components | 3.02 | 2.68 | 3.39 | <0.001 |

Model adjusted by age, sex, MEDEA Deprivation Index, smoking status, alcohol consumption and nationality

**Supplementary Table 10.** Prostate cancer. The combined effect of components of MS on prostate cancer incidence

|  | **Cancer risk** | | | |
| --- | --- | --- | --- | --- |
| **Metabolic syndrome** | **HR** | **95%** | **CI** | **Pvalue** |
| 0 components | 1.00 |  |  |  |
| 1 component | 1.36 | 1.31 | 1.42 | <0.001 |
| HDL | 1.41 | 1.19 | 1.67 | <0.001 |
| Glycemia | 1.70 | 1.59 | 1.83 | <0.001 |
| HBP | 1.31 | 1.25 | 1.37 | <0.001 |
| TG | 1.61 | 1.40 | 1.84 | <0.001 |
| Obesity | 1.08 | 0.90 | 1.29 | 0.424 |
| 2 components | 1.41 | 1.35 | 1.48 | <0.001 |
| HDL+Glycemia | 1.55 | 1.33 | 1.81 | <0.001 |
| HBP+Glycemia | 1.44 | 1.37 | 1.51 | <0.001 |
| HBP+HDL | 1.29 | 1.18 | 1.42 | <0.001 |
| HBP+TG | 1.45 | 1.33 | 1.57 | <0.001 |
| Glycemia+TG | 1.62 | 1.44 | 1.83 | <0.001 |
| Obesity+HDL | 1.52 | 0.96 | 2.42 | 0.076 |
| HDL+TG | 1.93 | 1.59 | 2.34 | <0.001 |
| HBP+ Obesity | 1.26 | 1.18 | 1.35 | <0.001 |
| Obesity +Glycemia | 1.28 | 1.07 | 1.54 | 0.007 |
| Obesity +TG | 1.37 | 0.92 | 2.05 | 0.123 |
| 3 components | 1.33 | 1.26 | 1.39 | <0.001 |
| HBP+HDL+Glycemia | 1.20 | 1.12 | 1.29 | <0.001 |
| HBP+Glycemia+TG | 1.41 | 1.32 | 1.50 | <0.001 |
| HBP+ Obesity +HDL | 1.24 | 1.07 | 1.44 | 0.003 |
| HBP+ Obesity +Glycemia | 1.33 | 1.25 | 1.41 | <0.001 |
| HBP+HDL+TG | 1.43 | 1.29 | 1.59 | <0.001 |
| HDL+Glycemia+TG | 1.33 | 1.12 | 1.58 | 0.001 |
| Obesity +HDL+Glycemia | 1.59 | 1.19 | 2.12 | 0.002 |
| HBP+ Obesity +TG | 1.21 | 1.06 | 1.38 | 0.004 |
| Obesity +HDL+TG | 1.36 | 0.79 | 2.34 | 0.272 |
| Obesity +Glycemia+TG | 0.80 | 0.58 | 1.11 | 0.184 |
| 4 components | 1.23 | 1.17 | 1.29 | <0.001 |
| HBP+HDL+Glycemia+TG | 1.26 | 1.18 | 1.34 | <0.001 |
| HBP+ Obesity +HDL+Glycemia | 1.10 | 1.02 | 1.19 | 0.019 |
| HBP+ Obesity +Glycemia+TG | 1.28 | 1.20 | 1.37 | <0.001 |
| HBP+ Obesity +HDL+TG | 1.11 | 0.95 | 1.30 | 0.182 |
| Obesity +HDL+Glycemia+TG | 1.24 | 0.92 | 1.67 | 0.150 |
| 5 components | 1.11 | 1.04 | 1.19 | 0.002 |

Model adjusted by age, sex, MEDEA Deprivation Index, smoking status, alcohol consumption and nationality

**Supplementary Table 11.** Hodgkin lymphoma. The combined effect of components of MS on Hodgkin lymphoma incidence

|  | **Cancer risk** | | | |
| --- | --- | --- | --- | --- |
| **Metabolic syndrome** | **HR** | **95%** | **CI** | **Pvalue** |
| 0 components | 1.00 |  |  |  |
| 1 component | 1.03 | 0.82 | 1.30 | 0.793 |
| HDL | 1.40 | 0.79 | 2.47 | 0.244 |
| Glycemia | 1.08 | 0.66 | 1.79 | 0.753 |
| HBP | 0.99 | 0.77 | 1.29 | 0.965 |
| TG | 0.88 | 0.36 | 2.16 | 0.788 |
| Obesity | 0.47 | 0.15 | 1.46 | 0.190 |
| 2 components | 1.40 | 1.09 | 1.79 | 0.009 |
| HDL+Glycemia | 4.04 | 2.41 | 6.76 | <0.001 |
| HBP+Glycemia | 1.18 | 0.83 | 1.68 | 0.365 |
| HBP+HDL | 2.19 | 1.44 | 3.32 | <0.001 |
| HBP+TG | 0.78 | 0.38 | 1.6 | 0.502 |
| Glycemia+TG | 0.28 | 0.04 | 2.03 | 0.210 |
| Obesity+HDL | 1.49 | 0.37 | 6.03 | 0.574 |
| HDL+TG | 2.31 | 1.13 | 4.70 | 0.021 |
| HBP+ Obesity | 0.98 | 0.62 | 1.54 | 0.923 |
| Obesity +Glycemia | 1.27 | 0.40 | 4.00 | 0.680 |
| Obesity +TG | --- | --- | --- | --- |
| 3 components | 1.50 | 1.14 | 1.98 | 0.004 |
| HBP+HDL+Glycemia | 1.80 | 1.16 | 2.79 | 0.009 |
| HBP+Glycemia+TG | 1.36 | 0.84 | 2.21 | 0.217 |
| HBP+ Obesity +HDL | 2.69 | 1.57 | 4.62 | <0.001 |
| HBP+ Obesity +Glycemia | 1.01 | 0.63 | 1.63 | 0.968 |
| HBP+HDL+TG | 1.47 | 0.79 | 2.74 | 0.222 |
| HDL+Glycemia+TG | 2.00 | 1.01 | 3.96 | 0.046 |
| Obesity +HDL+Glycemia | 2.32 | 0.74 | 7.28 | 0.151 |
| HBP+ Obesity +TG | 0.24 | 0.03 | 1.74 | 0.159 |
| Obesity +HDL+TG | 2.45 | 1.15 | 5.24 | 0.021 |
| Obesity +Glycemia+TG | 1.89 | 1.22 | 2.94 | 0.005 |
| 4 components | 1.83 | 1.36 | 2.47 | <0.001 |
| HBP+HDL+Glycemia+TG | 1.35 | 0.89 | 2.06 | 0.156 |
| HBP+ Obesity +HDL+Glycemia | 1.34 | 0.79 | 2.26 | 0.278 |
| HBP+ Obesity +Glycemia+TG | 1.64 | 0.40 | 6.63 | 0.489 |
| HBP+ Obesity +HDL+TG | 1.85 | 1.23 | 2.79 | 0.003 |
| Obesity +HDL+Glycemia+TG | 1.24 | 0.92 | 1.67 | 0.150 |
| 5 components | 1.43 | 0.94 | 2.18 | 0.091 |

Model adjusted by age, sex, MEDEA Deprivation Index, smoking status, alcohol consumption and nationality

**Supplementary Table 12.** Non-Hodgkin lymphoma. The combined effect of components of MS on non-Hodgkin lymphoma incidence

|  | **Cancer risk** | | | |
| --- | --- | --- | --- | --- |
| **Metabolic syndrome** | **HR** | **95%** | **CI** | **Pvalue** |
| 0 components | 1.00 |  |  |  |
| 1 component | 1.27 | 1.14 | 1.42 | <0.001 |
| HDL | 1.64 | 1.23 | 2.19 | 0.001 |
| Glycemia | 1.32 | 1.06 | 1.64 | 0.014 |
| HBP | 1.23 | 1.10 | 1.38 | <0.001 |
| TG | 0.74 | 0.44 | 1.23 | 0.243 |
| Obesity | 1.40 | 0.99 | 1.98 | 0.056 |
| 2 components | 1.33 | 1.18 | 1.50 | <0.001 |
| HDL+Glycemia | 1.73 | 1.18 | 2.53 | 0.005 |
| HBP+Glycemia | 1.19 | 1.02 | 1.38 | 0.025 |
| HBP+HDL | 2.21 | 1.83 | 2.68 | <0.001 |
| HBP+TG | 1.01 | 0.76 | 1.34 | 0.953 |
| Glycemia+TG | 1.25 | 0.80 | 1.95 | 0.336 |
| Obesity+HDL | 2.50 | 1.38 | 4.55 | 0.003 |
| HDL+TG | 1.32 | 0.80 | 2.16 | 0.277 |
| HBP+ Obesity | 1.10 | 0.91 | 1.33 | 0.325 |
| Obesity +Glycemia | 1.41 | 0.86 | 2.32 | 0.178 |
| Obesity +TG | 1.10 | 0.35 | 3.43 | 0.866 |
| 3 components | 1.39 | 1.22 | 1.57 | <0.001 |
| HBP+HDL+Glycemia | 1.91 | 1.59 | 2.29 | <0.001 |
| HBP+Glycemia+TG | 1.10 | 0.88 | 1.37 | 0.399 |
| HBP+ Obesity +HDL | 1.81 | 1.35 | 2.42 | <0.001 |
| HBP+ Obesity +Glycemia | 1.11 | 0.92 | 1.34 | 0.256 |
| HBP+HDL+TG | 1.36 | 1.02 | 1.80 | 0.037 |
| HDL+Glycemia+TG | 1.75 | 1.14 | 2.68 | 0.010 |
| Obesity +HDL+Glycemia | 1.36 | 0.64 | 2.86 | 0.422 |
| HBP+ Obesity +TG | 0.89 | 0.57 | 1.38 | 0.609 |
| Obesity +HDL+TG | 1.59 | 0.60 | 4.27 | 0.353 |
| Obesity +Glycemia+TG | 1.13 | 0.47 | 2.72 | 0.793 |
| 4 components | 1.73 | 1.51 | 1.97 | <0.001 |
| HBP+HDL+Glycemia+TG | 1.72 | 1.44 | 2.05 | <0.001 |
| HBP+ Obesity +HDL+Glycemia | 2.04 | 1.70 | 2.45 | <0.001 |
| HBP+ Obesity +Glycemia+TG | 1.26 | 1.02 | 1.57 | 0.036 |
| HBP+ Obesity +HDL+TG | 1.69 | 1.23 | 2.33 | 0.001 |
| Obesity +HDL+Glycemia+TG | 1.68 | 0.87 | 3.24 | 0.125 |
| 5 components | 1.70 | 1.44 | 2.00 | <0.001 |

Model adjusted by age, sex, MEDEA Deprivation Index, smoking status, alcohol consumption and nationality

**Supplementary Table 13.** Leukemia. The combined effect of components of MS on leukemia incidence

|  | **Cancer risk** | | | |
| --- | --- | --- | --- | --- |
| **Metabolic syndrome** | **HR** | **95%** | **CI** | **Pvalue** |
| 0 components | 1.00 |  |  |  |
| 1 component | 1.50 | 1.37 | 1.64 | <0.001 |
| HDL | 2.24 | 1.77 | 2.84 | <0.001 |
| Glycemia | 1.99 | 1.69 | 2.34 | <0.001 |
| HBP | 1.40 | 1.27 | 1.54 | <0.001 |
| TG | 1.36 | 0.97 | 1.92 | 0.078 |
| Obesity | 1.13 | 0.79 | 1.61 | 0.497 |
| 2 components | 1.73 | 1.57 | 1.90 | <0.001 |
| HDL+Glycemia | 3.12 | 2.42 | 4.03 | <0.001 |
| HBP+Glycemia | 1.69 | 1.51 | 1.88 | <0.001 |
| HBP+HDL | 2.18 | 1.86 | 2.55 | <0.001 |
| HBP+TG | 1.43 | 1.17 | 1.74 | <0.001 |
| Glycemia+TG | 1.49 | 1.05 | 2.11 | 0.026 |
| Obesity+HDL | 1.93 | 1.00 | 3.72 | 0.051 |
| HDL+TG | 1.12 | 0.68 | 1.84 | 0.657 |
| HBP+ Obesity | 1.50 | 1.31 | 1.73 | <0.001 |
| Obesity +Glycemia | 1.27 | 0.80 | 2.00 | 0.310 |
| Obesity +TG | 1.49 | 0.62 | 3.60 | 0.373 |
| 3 components | 1.90 | 1.72 | 2.10 | <0.001 |
| HBP+HDL+Glycemia | 2.79 | 2.46 | 3.17 | <0.001 |
| HBP+Glycemia+TG | 1.32 | 1.13 | 1.55 | 0.001 |
| HBP+ Obesity +HDL | 1.94 | 1.54 | 2.44 | <0.001 |
| HBP+ Obesity +Glycemia | 1.62 | 1.42 | 1.85 | <0.001 |
| HBP+HDL+TG | 2.24 | 1.86 | 2.70 | <0.001 |
| HDL+Glycemia+TG | 1.98 | 1.40 | 2.81 | <0.001 |
| Obesity +HDL+Glycemia | 1.47 | 0.76 | 2.84 | 0.249 |
| HBP+ Obesity +TG | 1.21 | 0.90 | 1.64 | 0.205 |
| Obesity +HDL+TG | 1.40 | 0.52 | 3.74 | 0.504 |
| Obesity +Glycemia+TG | 0.99 | 0.44 | 2.21 | 0.976 |
| 4 components | 2.17 | 1.96 | 2.41 | <0.001 |
| HBP+HDL+Glycemia+TG | 2.29 | 2.02 | 2.60 | <0.001 |
| HBP+ Obesity +HDL+Glycemia | 2.46 | 2.15 | 2.82 | <0.001 |
| HBP+ Obesity +Glycemia+TG | 1.59 | 1.36 | 1.86 | <0.001 |
| HBP+ Obesity +HDL+TG | 2.18 | 1.73 | 2.75 | <0.001 |
| Obesity +HDL+Glycemia+TG | 1.19 | 0.59 | 2.39 | 0.623 |
| 5 components | 2.30 | 2.04 | 2.59 | <0.001 |

Model adjusted by age, sex, MEDEA Deprivation Index, smoking status, alcohol consumption and nationality

**Supplementary Table 14.** Lung cancer. The combined effect of components of MS on lung cancer incidence

|  | **Cancer risk** | | | |
| --- | --- | --- | --- | --- |
| **Metabolic syndrome** | **HR** | **95%** | **CI** | **Pvalue** |
| 0 components | 1.00 |  |  |  |
| 1 component | 1.65 | 1.56 | 1.73 | <0.001 |
| HDL | 2.19 | 1.90 | 2.53 | <0.001 |
| Glycemia | 1.38 | 1.25 | 1.53 | <0.001 |
| HBP | 1.73 | 1.64 | 1.83 | <0.001 |
| TG | 1.11 | 0.91 | 1.35 | 0.321 |
| Obesity | 0.59 | 0.44 | 0.79 | <0.001 |
| 2 components | 1.78 | 1.69 | 1.88 | <0.001 |
| HDL+Glycemia | 2.05 | 1.73 | 2.43 | <0.001 |
| HBP+Glycemia | 2.00 | 1.88 | 2.12 | <0.001 |
| HBP+HDL | 2.29 | 2.08 | 2.52 | <0.001 |
| HBP+TG | 1.76 | 1.59 | 1.95 | <0.001 |
| Glycemia+TG | 1.19 | 0.98 | 1.44 | 0.087 |
| Obesity+HDL | 1.04 | 0.59 | 1.83 | 0.893 |
| HDL+TG | 1.36 | 1.06 | 1.74 | 0.016 |
| HBP+ Obesity | 1.31 | 1.20 | 1.43 | <0.001 |
| Obesity +Glycemia | 0.90 | 0.66 | 1.22 | 0.502 |
| Obesity +TG | 0.38 | 0.14 | 1.01 | 0.052 |
| 3 components | 1.77 | 1.67 | 1.87 | <0.001 |
| HBP+HDL+Glycemia | 2.48 | 2.3 | 2.67 | <0.001 |
| HBP+Glycemia+TG | 1.91 | 1.77 | 2.06 | <0.001 |
| HBP+ Obesity +HDL | 1.41 | 1.19 | 1.68 | <0.001 |
| HBP+ Obesity +Glycemia | 1.44 | 1.33 | 1.56 | <0.001 |
| HBP+HDL+TG | 1.99 | 1.77 | 2.23 | <0.001 |
| HDL+Glycemia+TG | 1.43 | 1.16 | 1.77 | 0.001 |
| Obesity +HDL+Glycemia | 0.97 | 0.62 | 1.52 | 0.892 |
| HBP+ Obesity +TG | 1.17 | 0.98 | 1.40 | 0.083 |
| Obesity +HDL+TG | 0.72 | 0.32 | 1.61 | 0.425 |
| Obesity +Glycemia+TG | 0.93 | 0.60 | 1.42 | 0.726 |
| 4 components | 1.80 | 1.70 | 1.91 | <0.001 |
| HBP+HDL+Glycemia+TG | 2.24 | 2.08 | 2.41 | <0.001 |
| HBP+ Obesity +HDL+Glycemia | 1.82 | 1.67 | 1.99 | <0.001 |
| HBP+ Obesity +Glycemia+TG | 1.44 | 1.32 | 1.57 | <0.001 |
| HBP+ Obesity +HDL+TG | 1.44 | 1.21 | 1.71 | <0.001 |
| Obesity +HDL+Glycemia+TG | 0.90 | 0.59 | 1.39 | 0.646 |
| 5 components | 1.71 | 1.59 | 1.84 | <0.001 |

Model adjusted by age, sex, MEDEA Deprivation Index, smoking status, alcohol consumption and nationality

**Supplementary Table 15.** Thyroid cancer. The combined effect of components of MS on thyroid cancer incidence

|  | **Cancer risk** | | | |
| --- | --- | --- | --- | --- |
| **Metabolic syndrome** | **HR** | **95%** | **CI** | **Pvalue** |
| 0 components | 1.00 |  |  |  |
| 1 component | 1.38 | 1.23 | 1.55 | <0.001 |
| HDL | 1.44 | 1.11 | 1.88 | 0.007 |
| Glycemia | 1.66 | 1.33 | 2.08 | <0.001 |
| HBP | 1.34 | 1.19 | 1.52 | <0.001 |
| TG | 1.11 | 0.70 | 1.75 | 0.660 |
| Obesity | 1.14 | 0.80 | 1.63 | 0.459 |
| 2 components | 1.67 | 1.47 | 1.89 | <0.001 |
| HDL+Glycemia | 2.30 | 1.62 | 3.26 | <0.001 |
| HBP+Glycemia | 1.46 | 1.21 | 1.75 | <0.001 |
| HBP+HDL | 2.30 | 1.86 | 2.83 | <0.001 |
| HBP+TG | 1.22 | 0.87 | 1.70 | 0.256 |
| Glycemia+TG | 1.64 | 0.98 | 2.74 | 0.059 |
| Obesity+HDL | 2.02 | 1.31 | 3.67 | 0.003 |
| HDL+TG | 1.90 | 1.26 | 2.86 | 0.002 |
| HBP+ Obesity | 1.43 | 1.17 | 1.75 | <0.001 |
| Obesity +Glycemia | 2.00 | 1.27 | 3.17 | 0.003 |
| Obesity +TG | 1.58 | 0.59 | 4.24 | 0.360 |
| 3 components | 1.70 | 1.47 | 1.95 | <0.001 |
| HBP+HDL+Glycemia | 1.68 | 1.30 | 2.17 | <0.001 |
| HBP+Glycemia+TG | 1.50 | 1.12 | 1.99 | 0.006 |
| HBP+ Obesity +HDL | 1.52 | 1.09 | 2.13 | 0.014 |
| HBP+ Obesity +Glycemia | 1.55 | 1.24 | 1.92 | <0.001 |
| HBP+HDL+TG | 1.90 | 1.41 | 2.55 | <0.001 |
| HDL+Glycemia+TG | 2.00 | 1.27 | 3.16 | 0.003 |
| Obesity +HDL+Glycemia | 1.80 | 0.96 | 3.37 | 0.065 |
| HBP+ Obesity +TG | 2.03 | 1.39 | 2.97 | <0.001 |
| Obesity +HDL+TG | 2.48 | 1.24 | 5.00 | 0.011 |
| Obesity +Glycemia+TG | 1.04 | 0.34 | 3.25 | 0.941 |
| 4 components | 1.96 | 1.68 | 2.28 | <0.001 |
| HBP+HDL+Glycemia+TG | 1.92 | 1.53 | 2.42 | <0.001 |
| HBP+ Obesity +HDL+Glycemia | 1.93 | 1.53 | 2.42 | <0.001 |
| HBP+ Obesity +Glycemia+TG | 1.87 | 1.44 | 2.42 | <0.001 |
| HBP+ Obesity +HDL+TG | 1.92 | 1.36 | 2.71 | <0.001 |
| Obesity +HDL+Glycemia+TG | 1.96 | 1.05 | 3.67 | 0.035 |
| 5 components | 1.78 | 1.46 | 2.17 | <0.001 |

Model adjusted by age, sex, MEDEA Deprivation Index, smoking status, alcohol consumption and nationality

**Supplementary Table 16**. Others cancer. The combined effect of components of MS on others cancer incidence

|  | **Cancer risk** | | | |
| --- | --- | --- | --- | --- |
| **Metabolic syndrome** | **HR** | **95%** | **CI** | **Pvalue** |
| 0 components | 1.00 |  |  |  |
| 1 component | 1.29 | 1.26 | 1.31 | <0.001 |
| HDL | 1.49 | 1.41 | 1.58 | <0.001 |
| Glycemia | 1.36 | 1.31 | 1.42 | <0.001 |
| HBP | 1.28 | 1.25 | 1.31 | <0.001 |
| TG | 1.27 | 1.18 | 1.37 | <0.001 |
| Obesity | 1.06 | 0.99 | 1.15 | 0.113 |
| 2 components | 1.40 | 1.37 | 1.43 | <0.001 |
| HDL+Glycemia | 1.40 | 1.30 | 1.52 | <0.001 |
| HBP+Glycemia | 1.47 | 1.44 | 1.51 | <0.001 |
| HBP+HDL | 1.54 | 1.48 | 1.60 | <0.001 |
| HBP+TG | 1.38 | 1.32 | 1.44 | <0.001 |
| Glycemia+TG | 1.26 | 1.16 | 1.36 | <0.001 |
| Obesity+HDL | 1.24 | 1.06 | 1.46 | 0.009 |
| HDL+TG | 1.38 | 1.26 | 1.51 | <0.001 |
| HBP+ Obesity | 1.22 | 1.19 | 1.26 | <0.001 |
| Obesity +Glycemia | 1.12 | 1.01 | 1.24 | 0.029 |
| Obesity +TG | 0.96 | 0.77 | 1.20 | 0.705 |
| 3 components | 1.40 | 1.37 | 1.44 | <0.001 |
| HBP+HDL+Glycemia | 1.55 | 1.50 | 1.60 | <0.001 |
| HBP+Glycemia+TG | 1.45 | 1.40 | 1.50 | <0.001 |
| HBP+ Obesity +HDL | 1.43 | 1.35 | 1.50 | <0.001 |
| HBP+ Obesity +Glycemia | 1.33 | 1.29 | 1.37 | <0.001 |
| HBP+HDL+TG | 1.45 | 1.38 | 1.51 | <0.001 |
| HDL+Glycemia+TG | 1.27 | 1.16 | 1.39 | <0.001 |
| Obesity +HDL+Glycemia | 1.12 | 0.96 | 1.31 | 0.136 |
| HBP+ Obesity +TG | 1.31 | 1.24 | 1.39 | <0.001 |
| Obesity +HDL+TG | 0.97 | 0.77 | 1.22 | 0.807 |
| Obesity +Glycemia+TG | 0.88 | 0.74 | 1.04 | 0.142 |
| 4 components | 1.45 | 1.42 | 1.49 | <0.001 |
| HBP+HDL+Glycemia+TG | 1.54 | 1.49 | 1.58 | <0.001 |
| HBP+ Obesity +HDL+Glycemia | 1.45 | 1.40 | 1.50 | <0.001 |
| HBP+ Obesity +Glycemia+TG | 1.39 | 1.34 | 1.43 | <0.001 |
| HBP+ Obesity +HDL+TG | 1.42 | 1.35 | 1.50 | <0.001 |
| Obesity +HDL+Glycemia+TG | 1.13 | 0.98 | 1.30 | 0.104 |
| 5 components | 1.45 | 1.41 | 1.49 | <0.001 |

Model adjusted by age, sex, MEDEA Deprivation Index, smoking status, alcohol consumption and nationality

**Supplementary Figure 1**. Overall cancer incidence (matched by age and sex) (The dashed lines represent the 95% CIs)


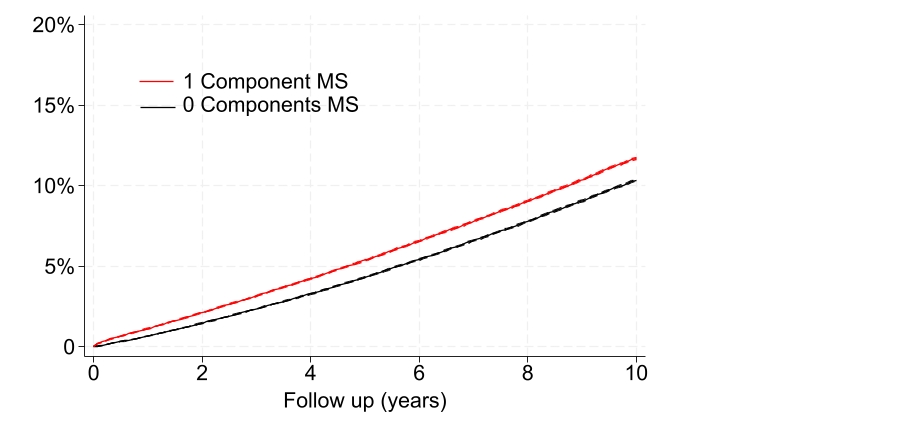

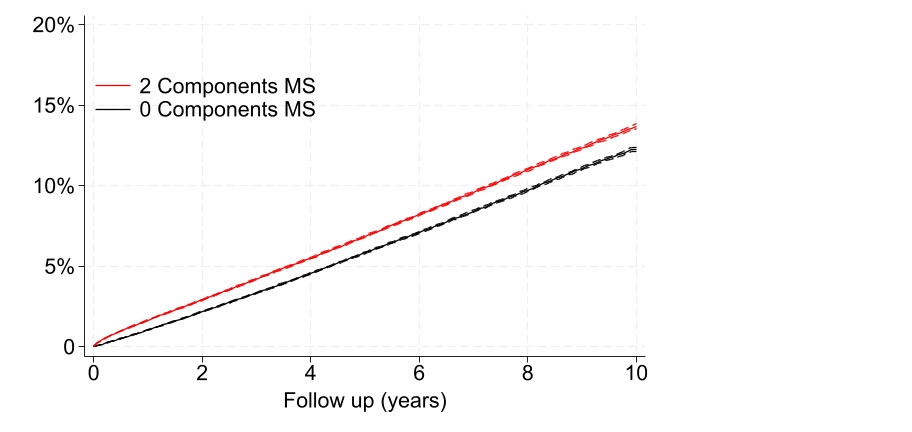

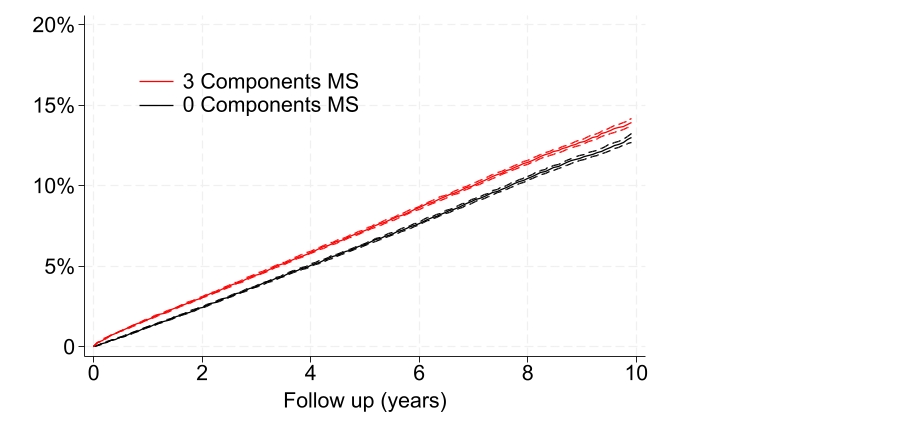


**Supplementary Figure 2.** Colorectal cancer incidence (matched by age and sex) (The dashed lines represent the 95% CIs)


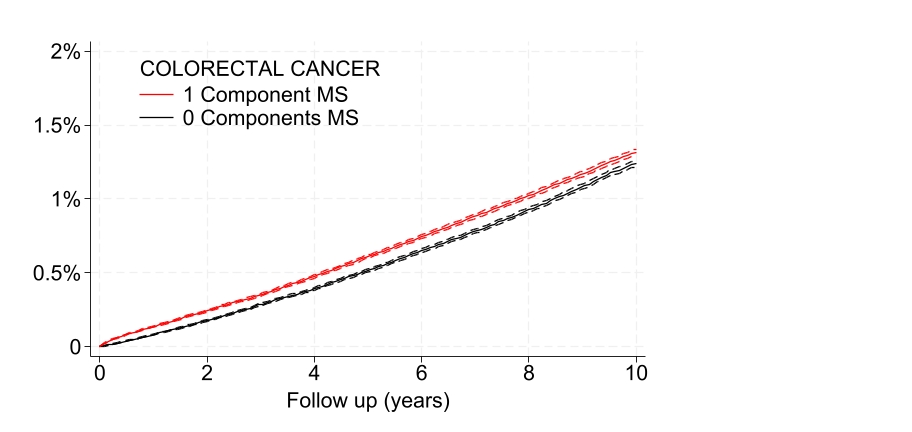

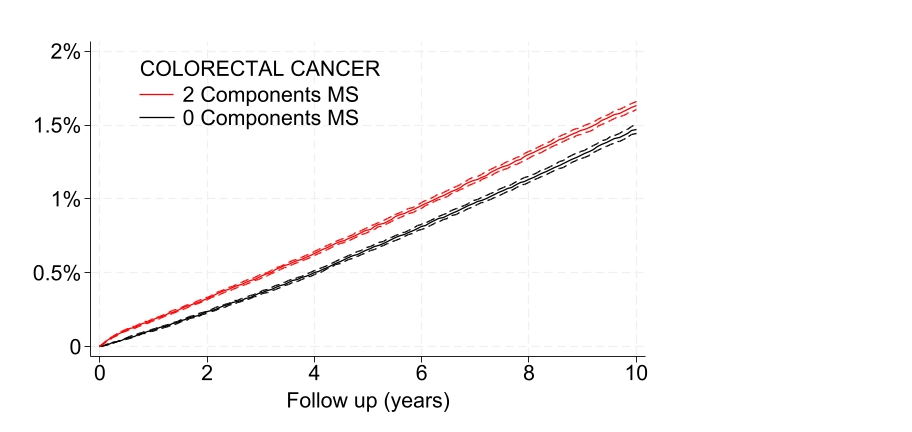

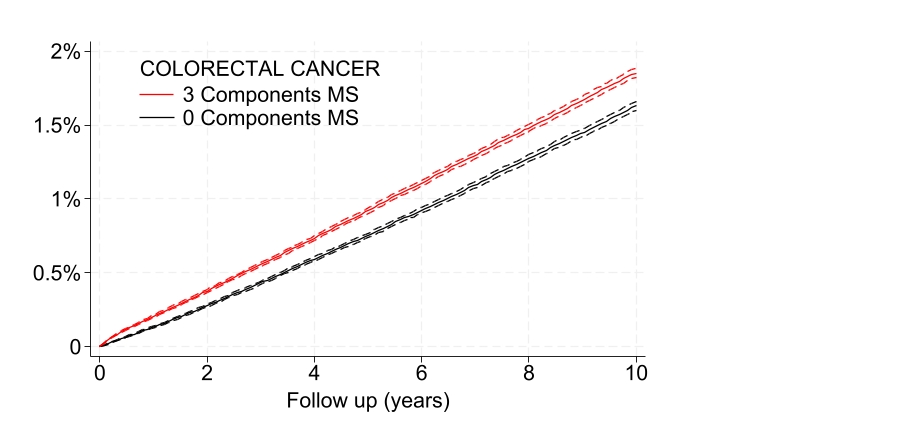


**Supplementary Figure 3**. Liver cancer incidence (matched by age and sex) (The dashed lines represent the 95% CIs)


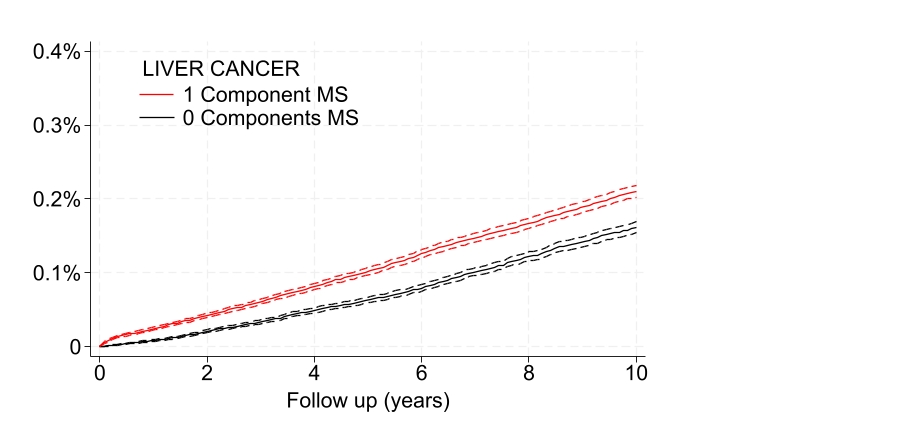

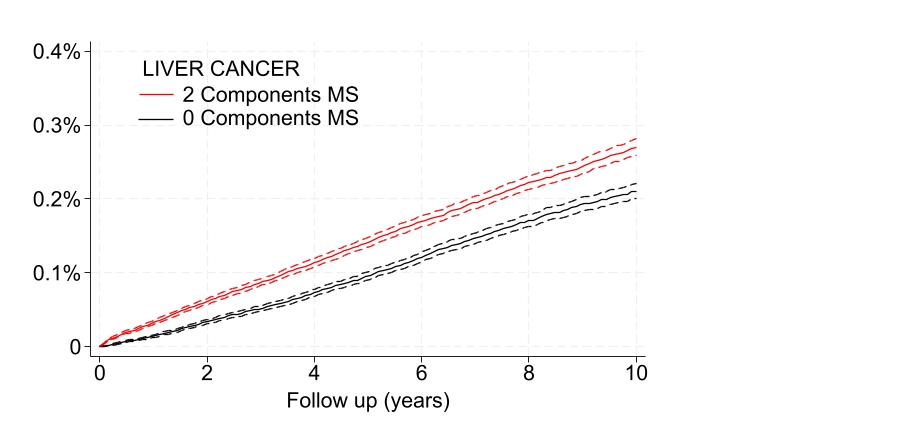

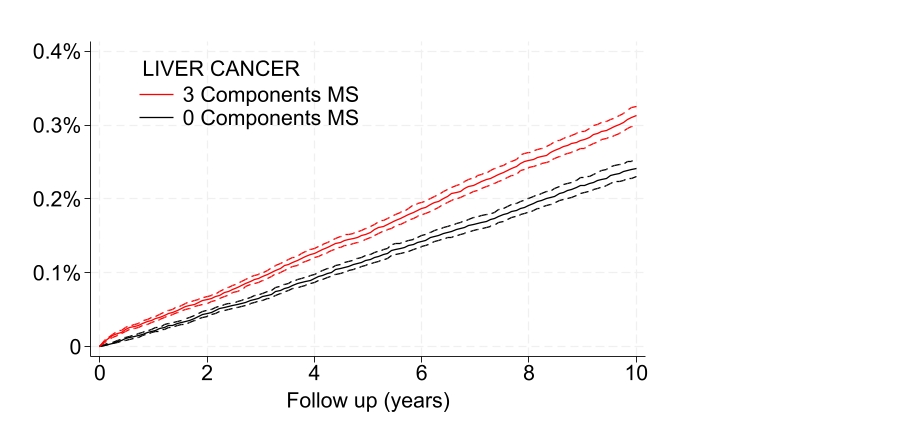


**Supplementary Figure 4.** Pancreas cancer incidence (matched by age and age and sex) (The dashed lines represent the 95% CIs)


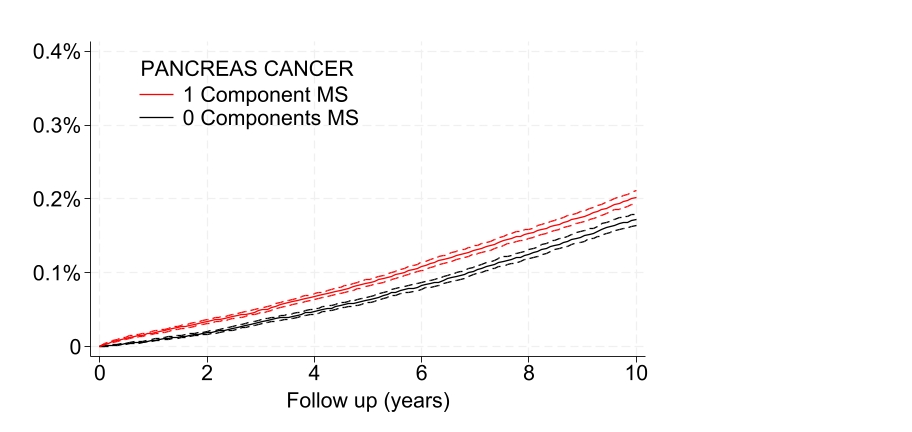

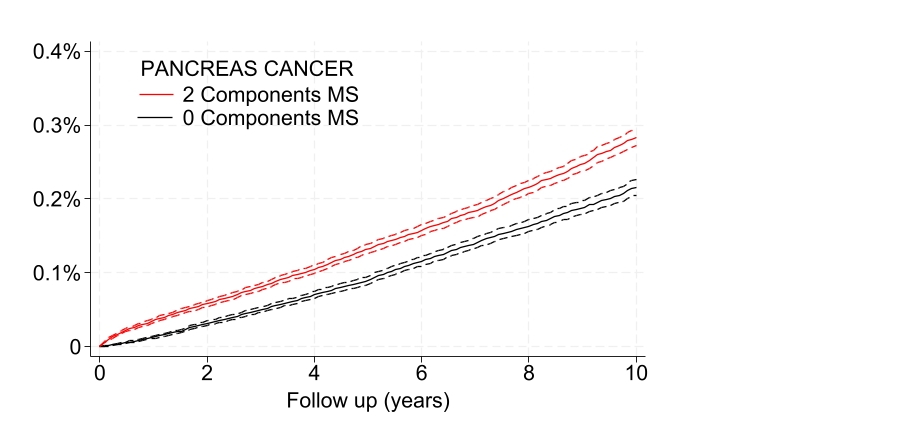

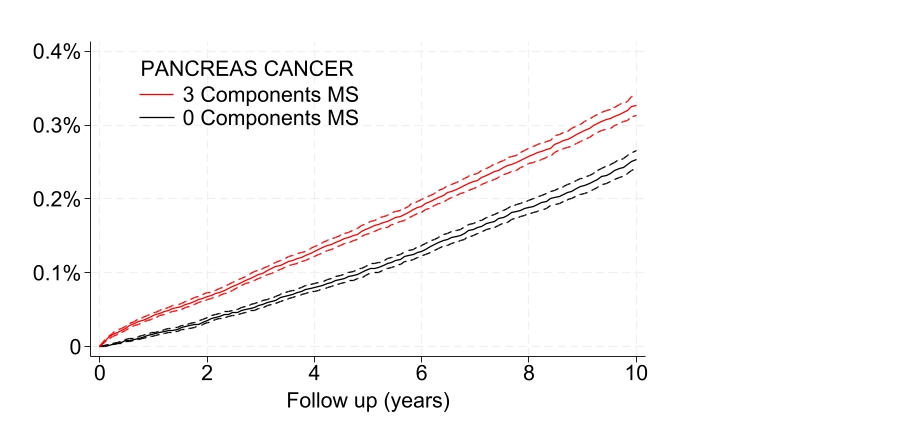


**Supplementary Figure 5**. Pre-menopause breast cancer incidence (matched by age) (The dashed lines represent the 95% CIs)


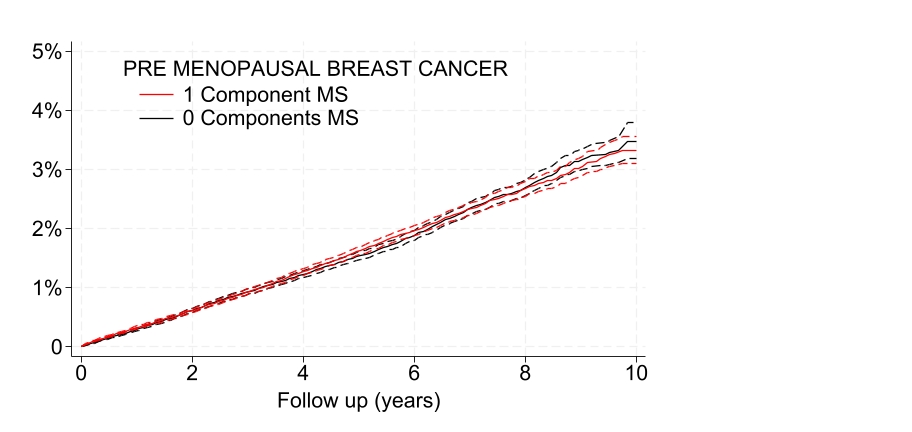

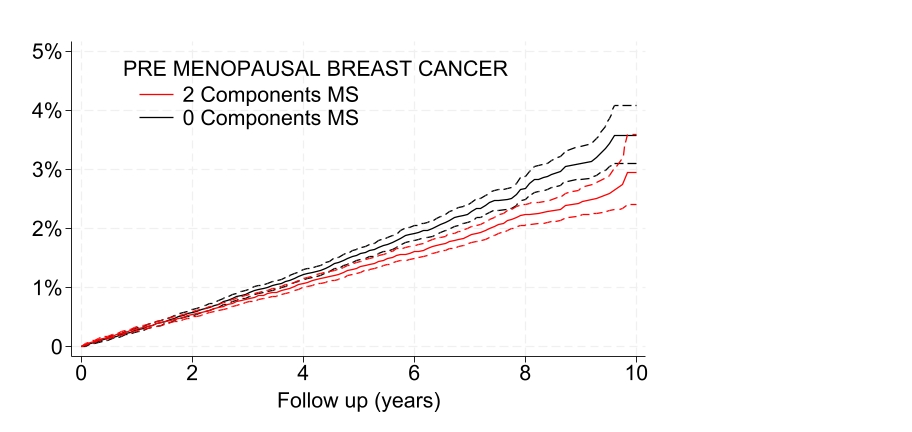

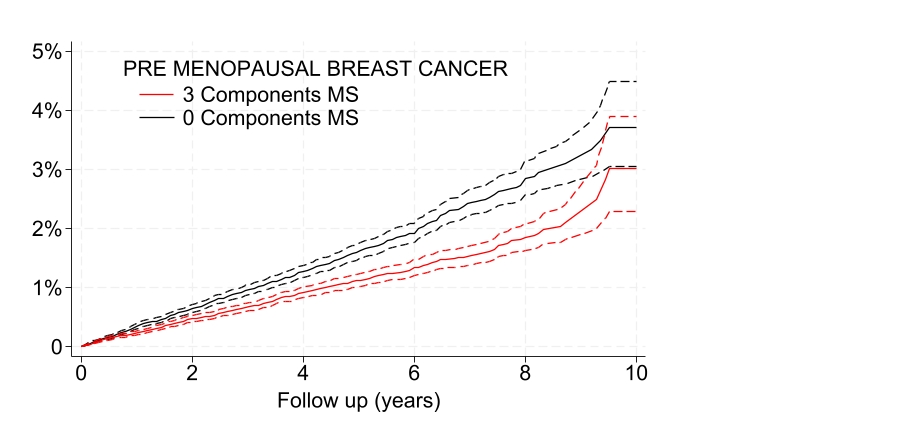


**Supplementary Figure 6.** Post menopause Breast cancer incidence (matched by age) (The dashed lines represent the 95% CIs)


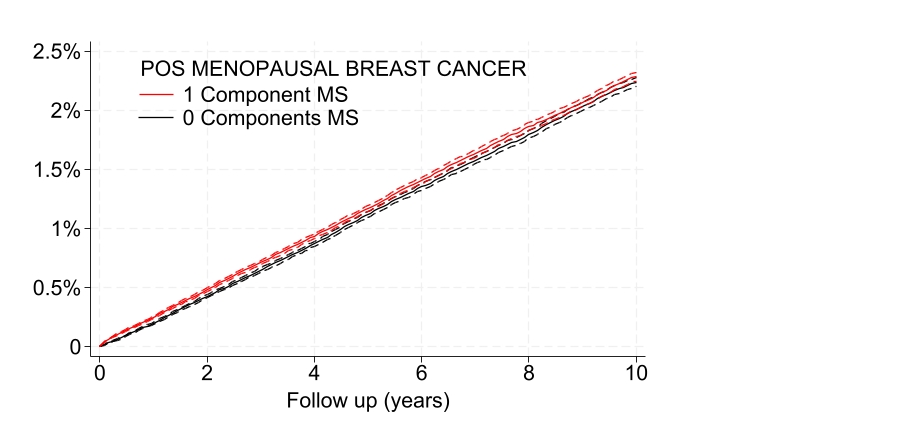

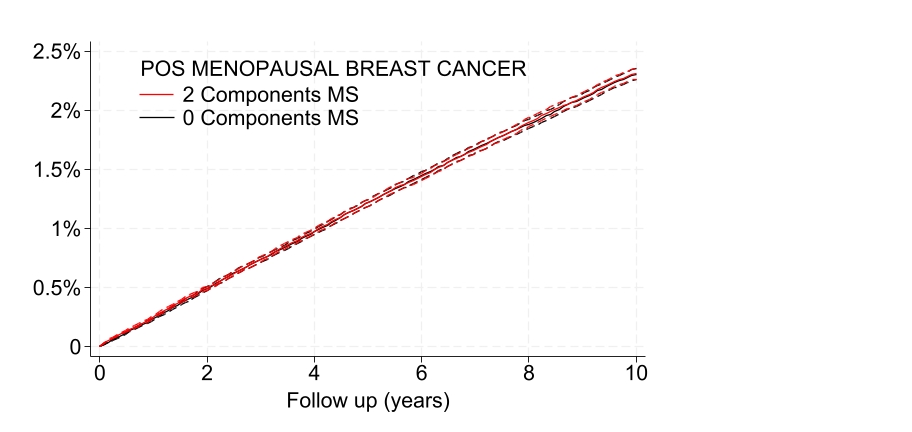

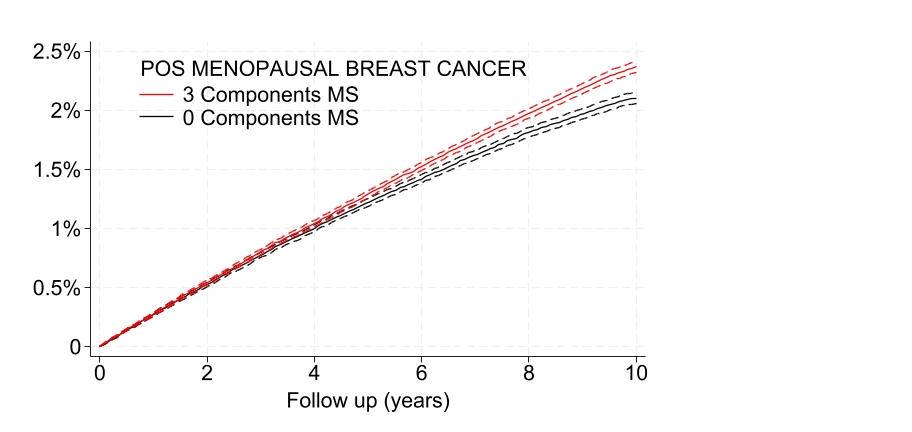


**Supplementary Figure 7**. Pre-menopause endometrial cancer incidence (matched by age) (The dashed lines represent the 95% CIs)


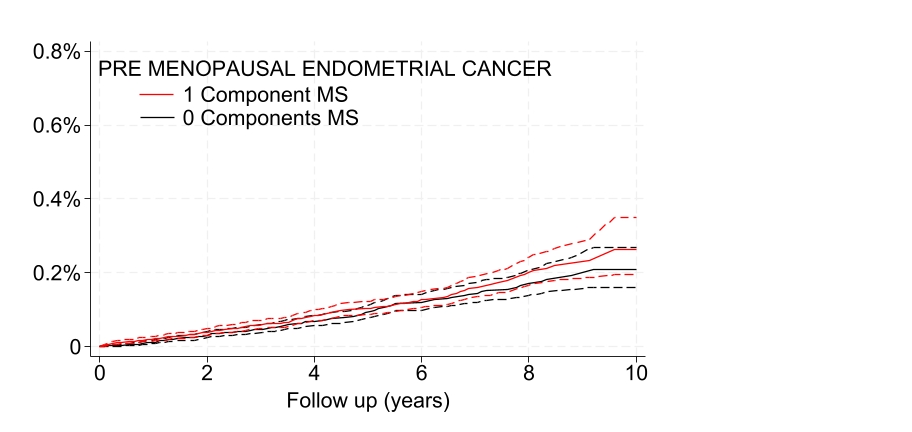

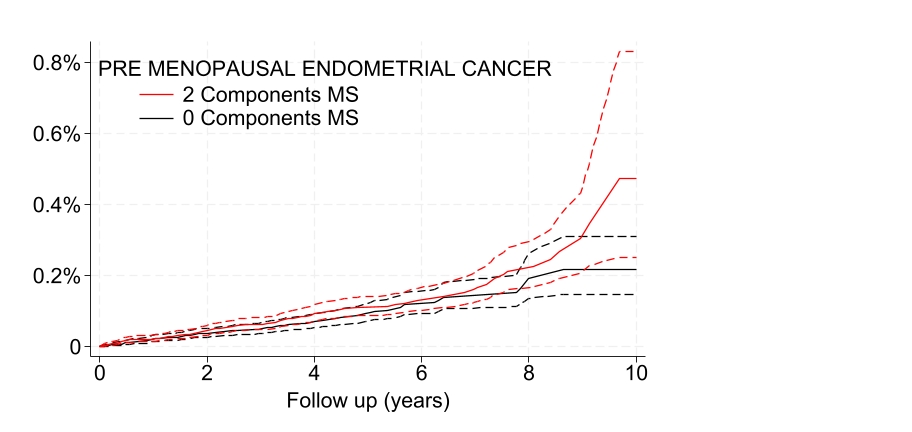

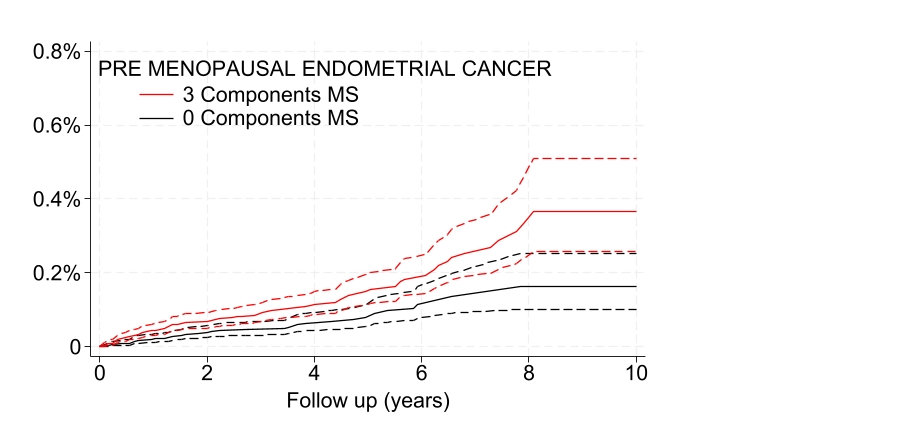


**Supplementary Figure 8**. Post-menopause endometrial cancer incidence (matched by age) (The dashed lines represent the 95% CIs)


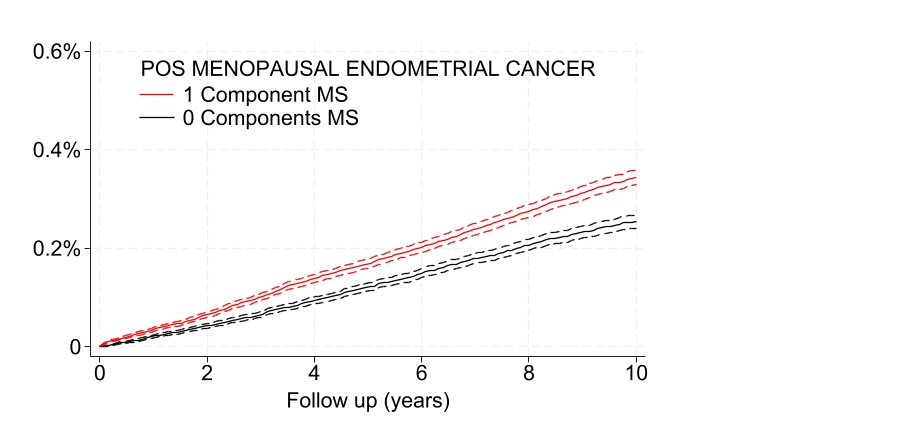

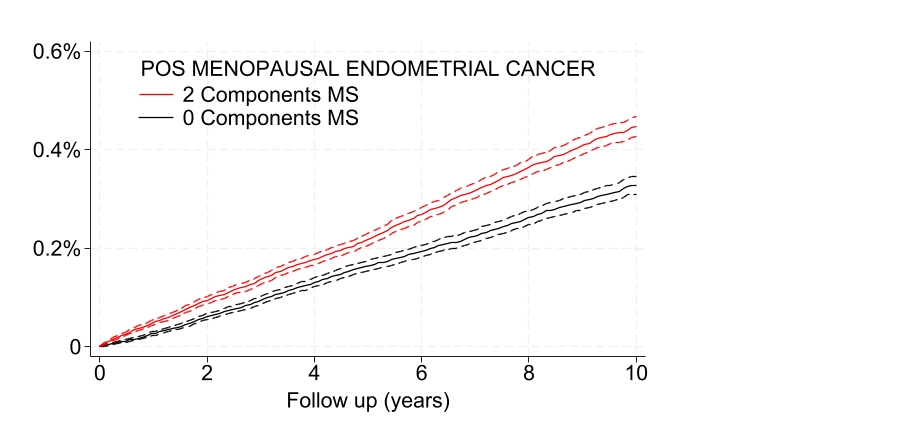

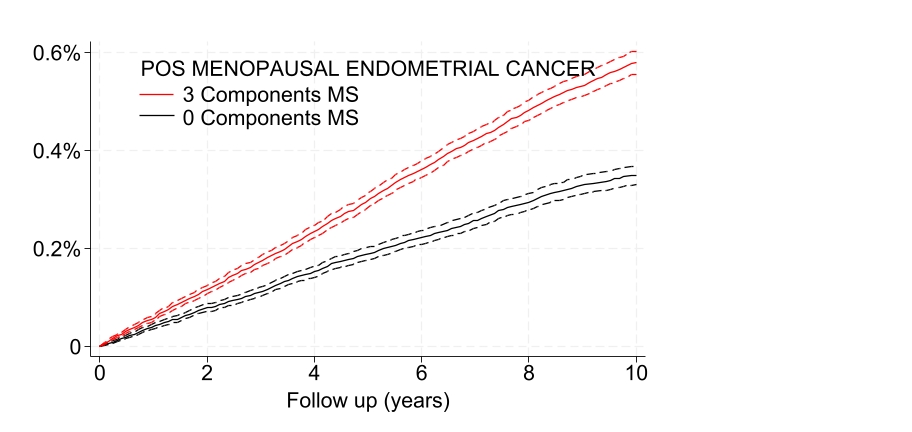


**Supplementary Figure 9.** Bladder cancer incidence (matched by age and sex) (The dashed lines represent the 95% CIs)


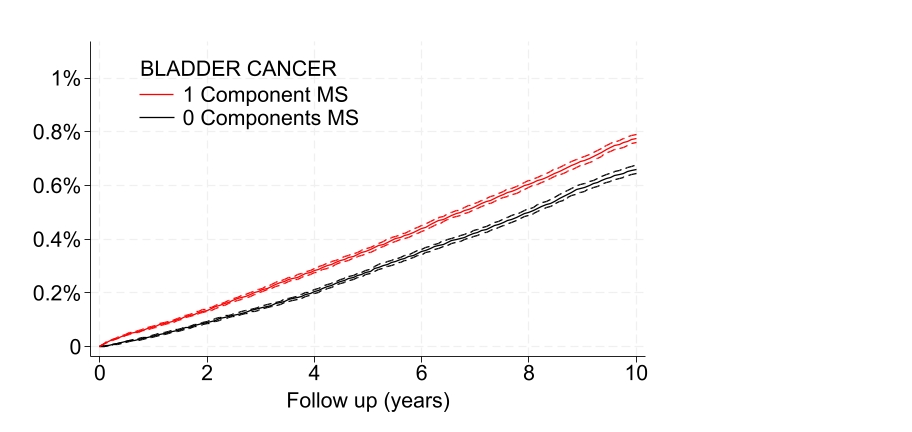

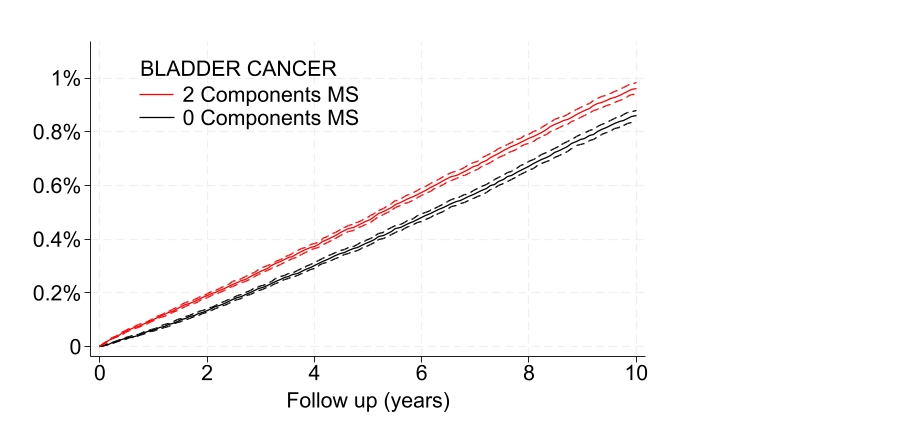

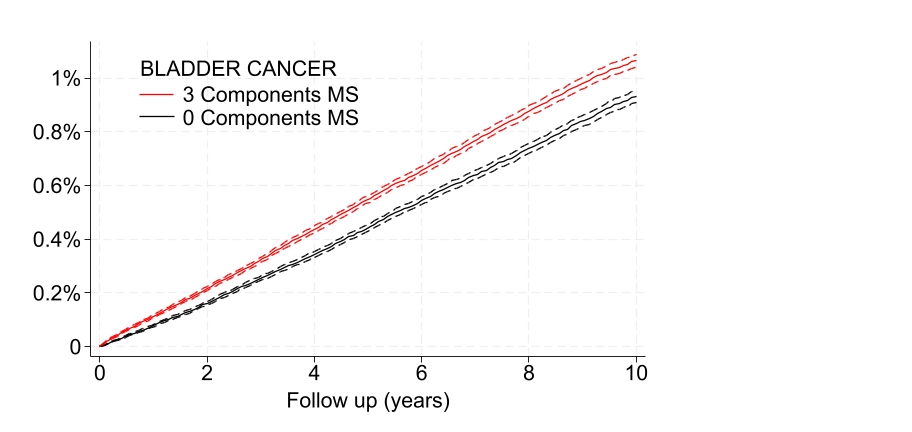


**Supplementary Figure 10.** Kidney cancer incidence (matched by age and sex) (The dashed lines represent the 95% CIs)


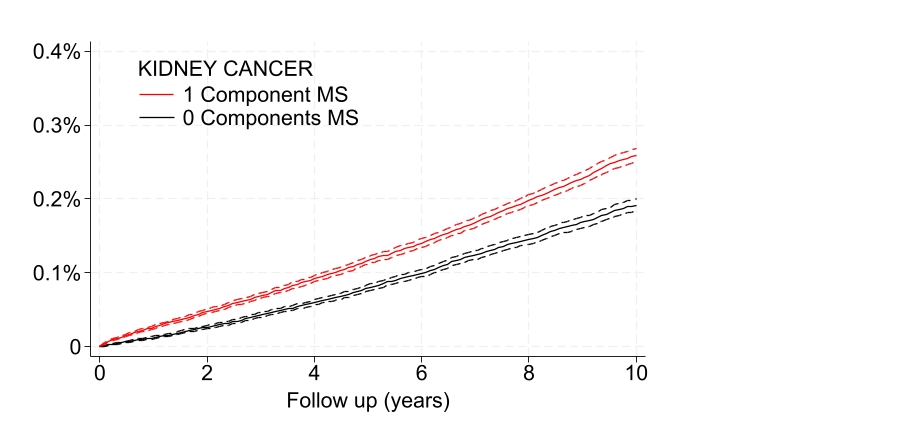

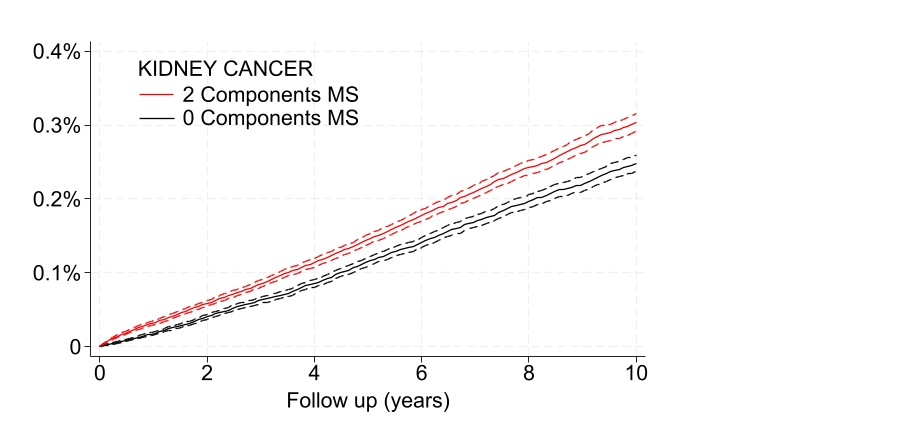

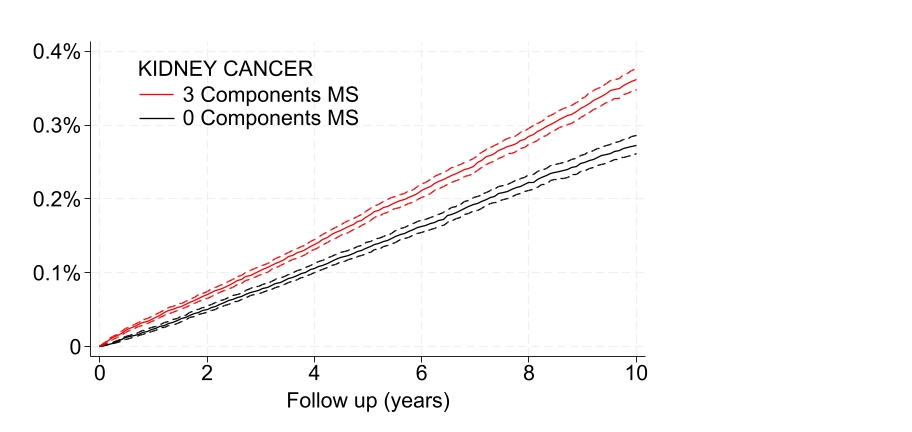


**Supplementary Figure 11**. Prostate cancer incidence (matched by age) (The dashed lines represent the 95% CIs)


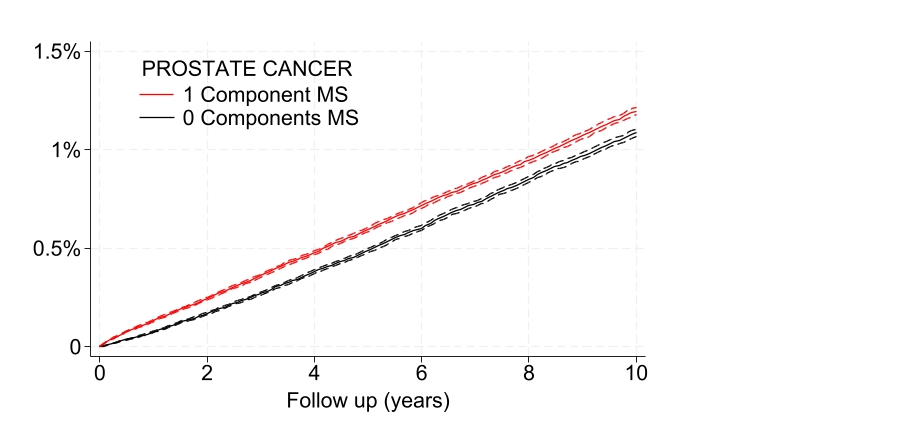

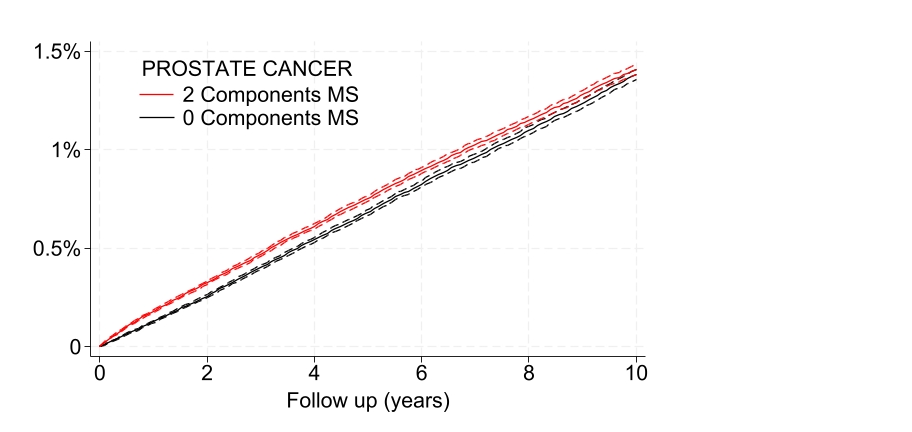

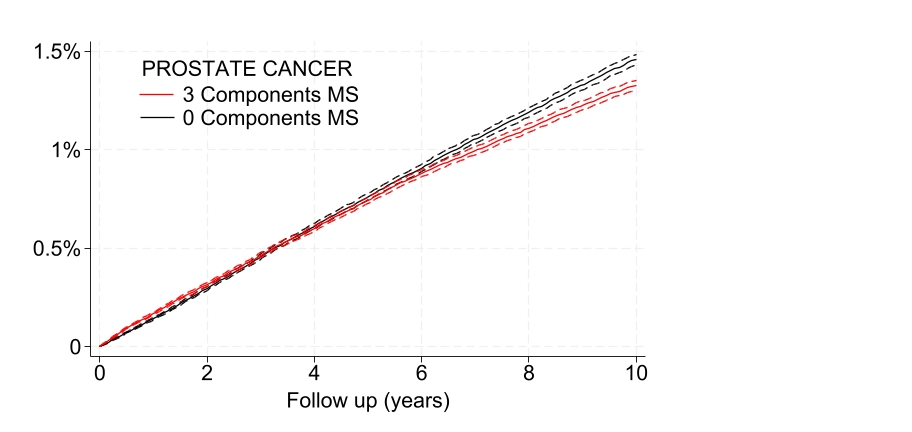


**Supplementary Figure 12.** Hodgkin lymphoma incidence (matched by age and sex) (The dashed lines represent the 95% CIs)


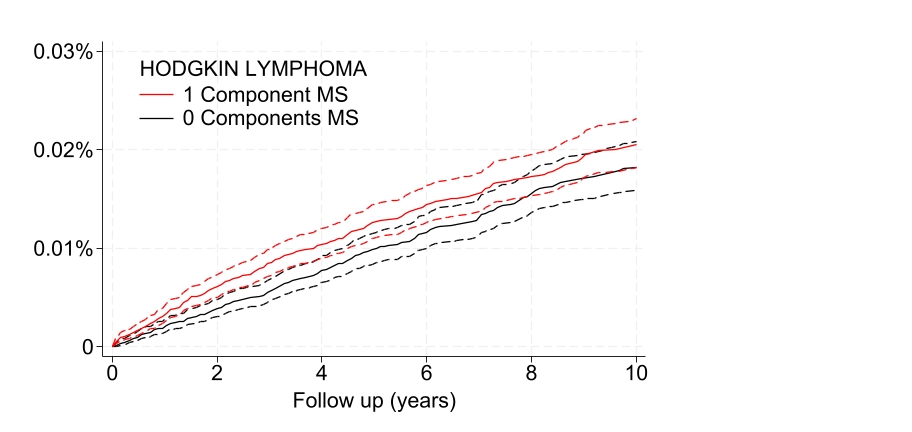

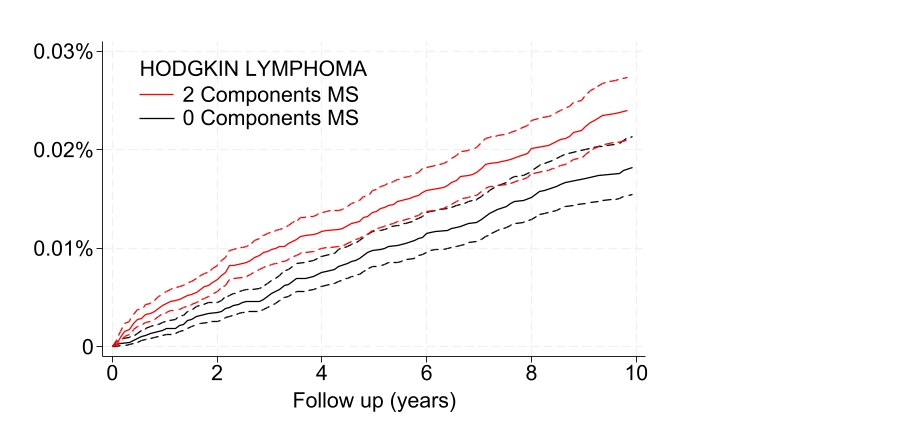

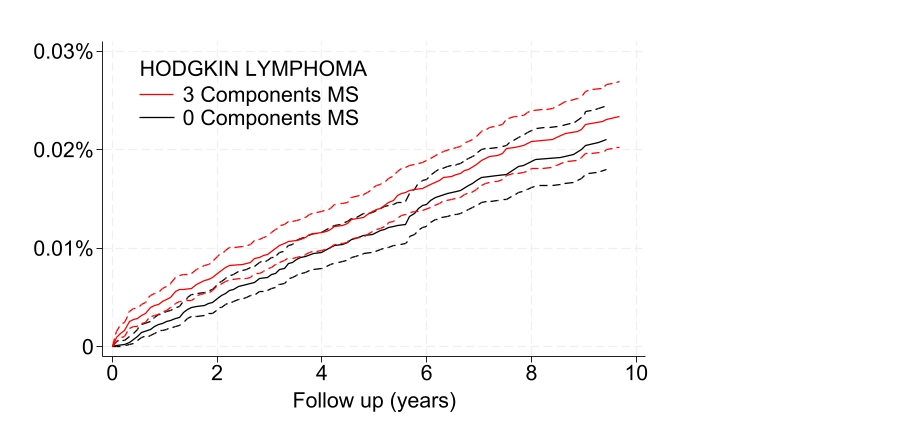


**Supplementary Figure 13.** Non-Hodgkin lymphoma incidence (matched by age and sex) (The dashed lines represent the 95% CIs)


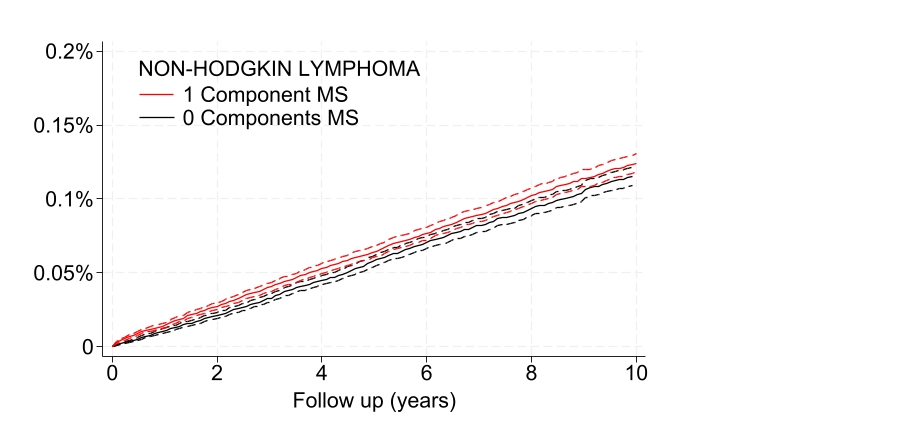

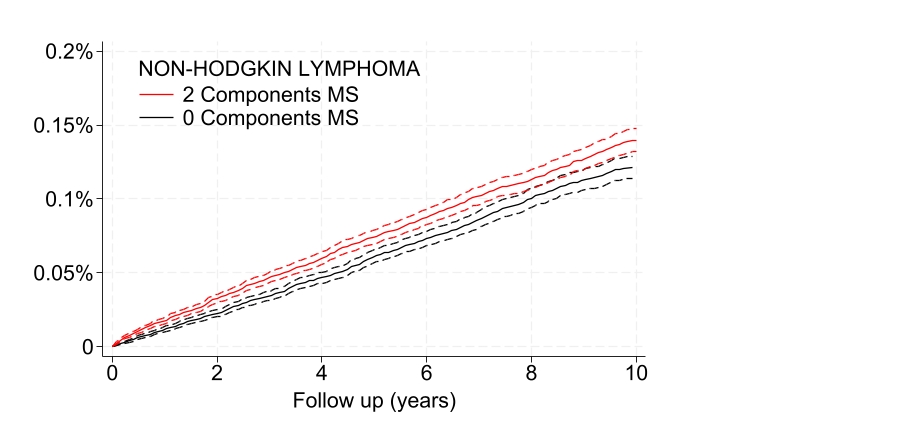

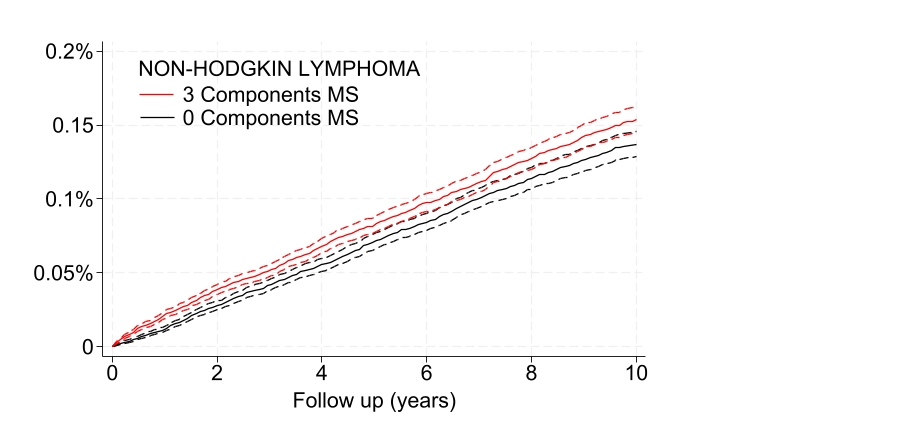


**Supplementary Figure 14.** Leukemia incidence (matched by age and sex) (The dashed lines represent the 95% CIs)


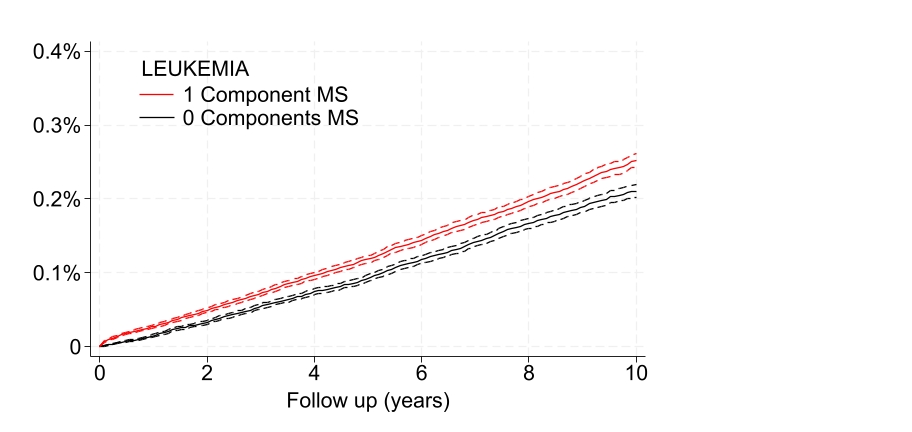

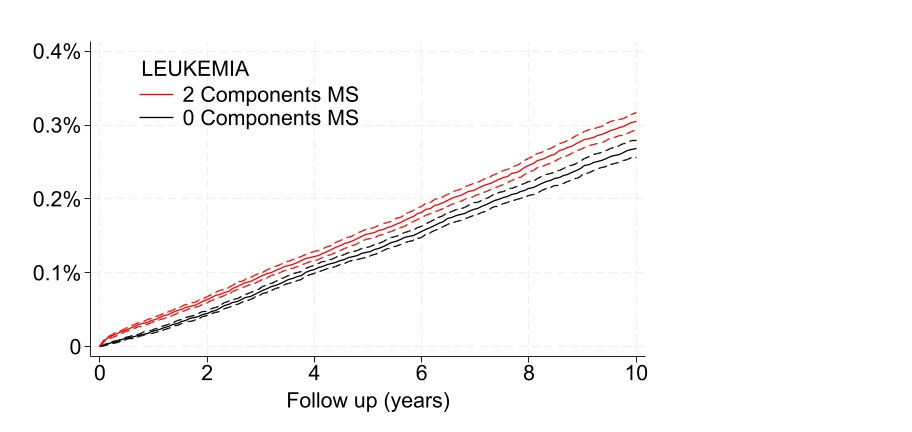

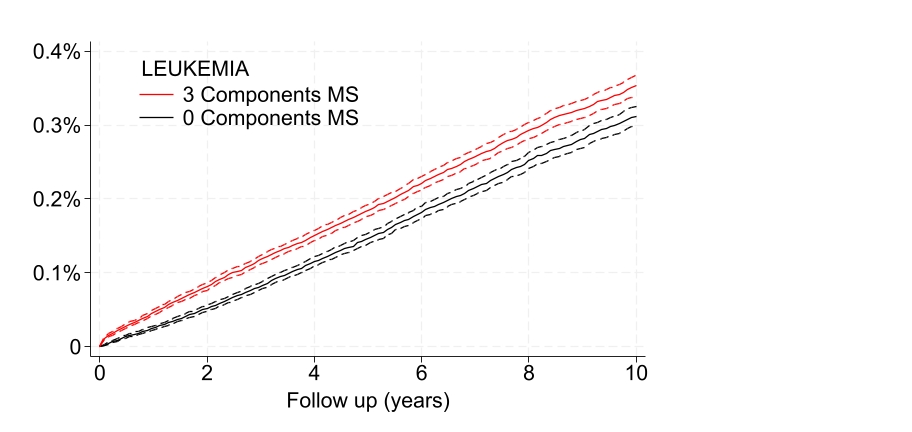


**Supplementary Figure 15**. Lung cancer incidence (matched by age and sex) (The dashed lines represent the 95% CIs)


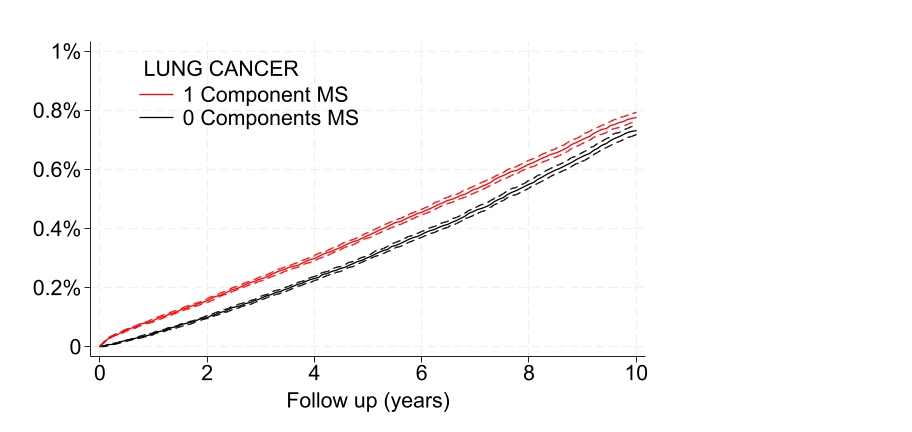

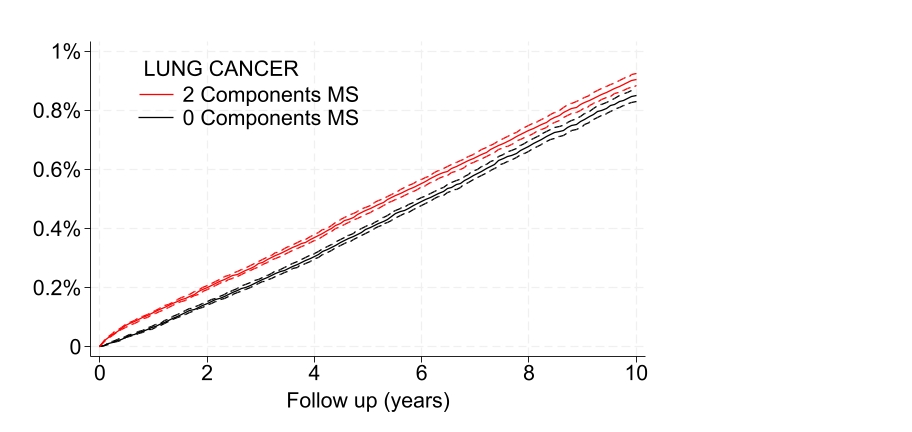

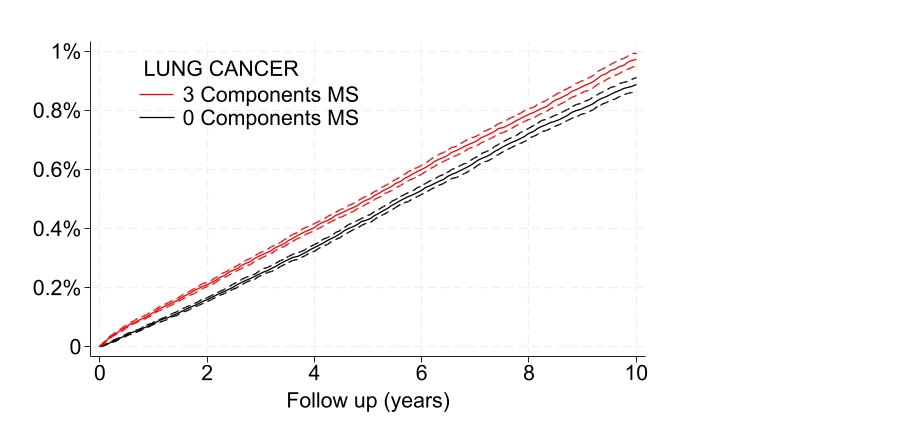


**Supplementary Figure 16**. Thyroid cancer incidence (matched by age and sex) (The dashed lines represent the 95% CIs)


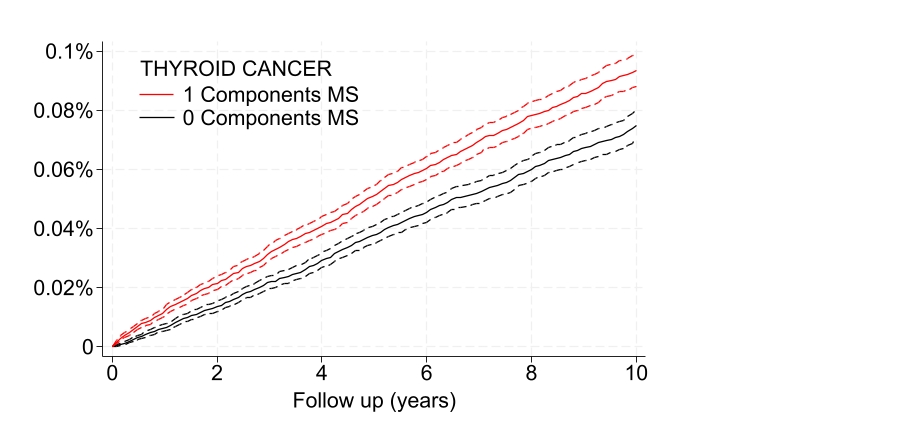

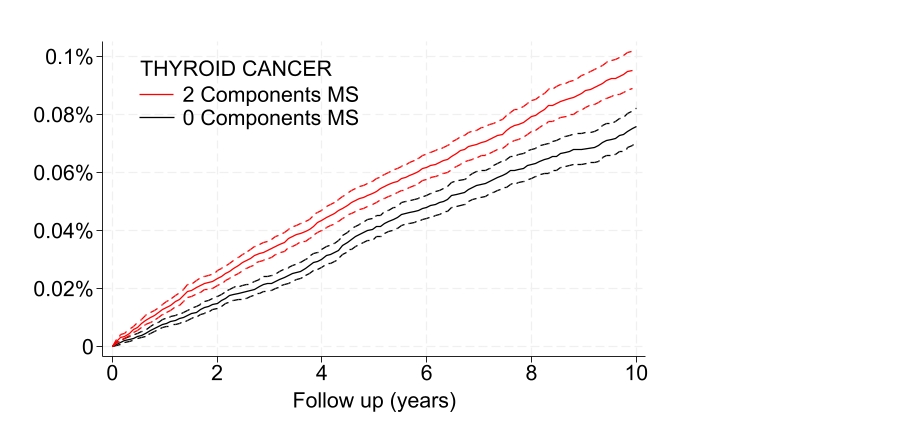

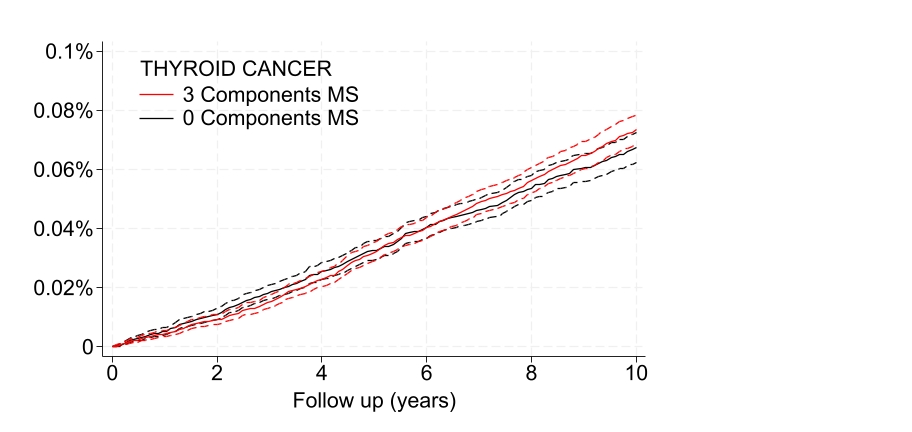


**Supplementary Figure 17.** Other cancer incidence (matched by age and sex) (The dashed lines represent the 95% CIs)


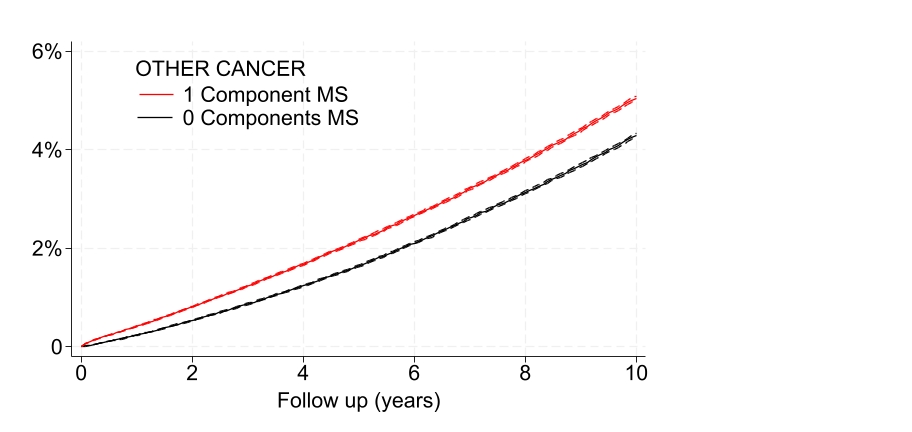

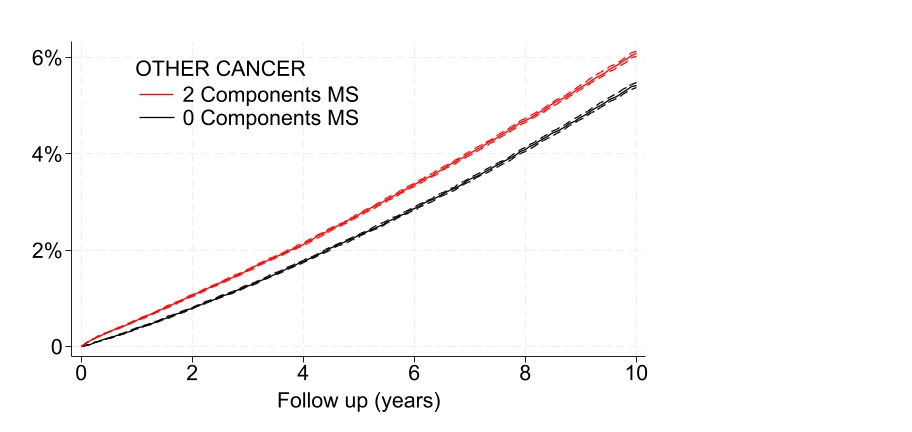

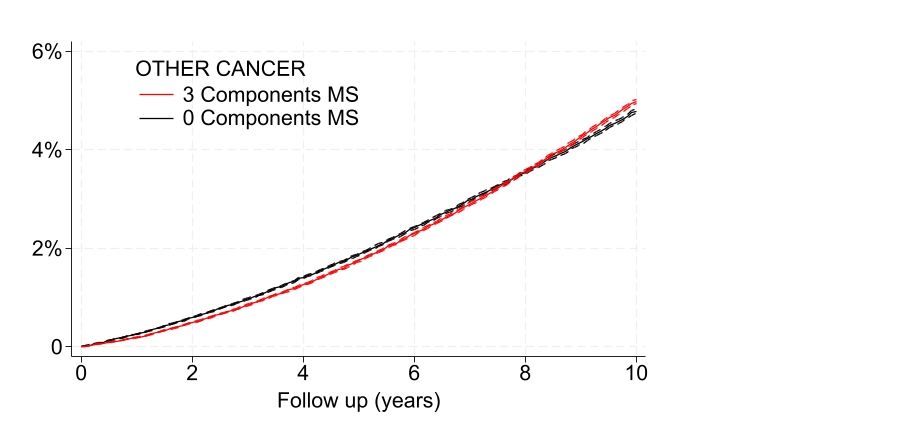


**Supplementary Figure 18.** Cumulative incidence function curves for free survival cancer stratified by individuals with components of MS, compared with individuals with no MS, matched by age and stratified by sex.


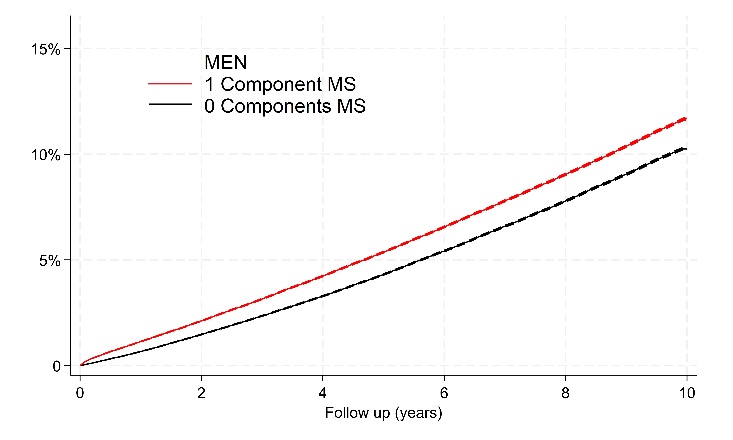

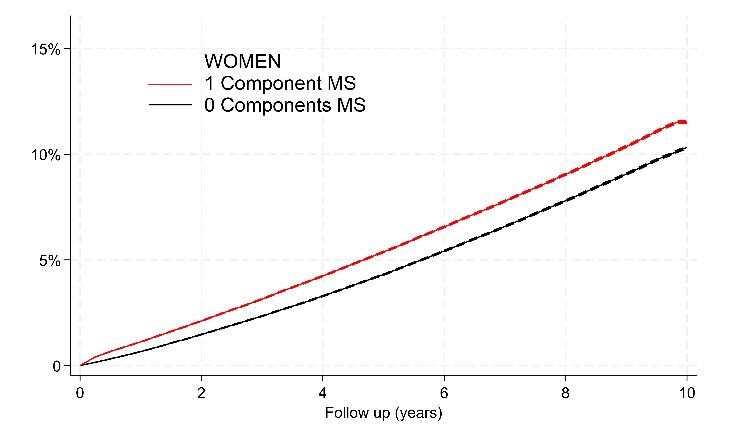

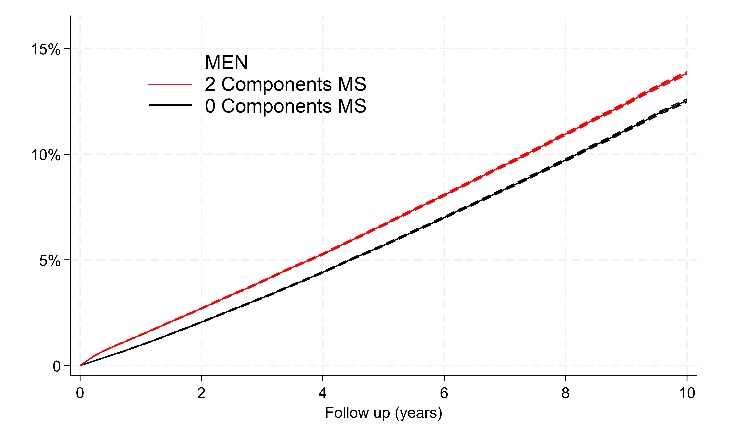

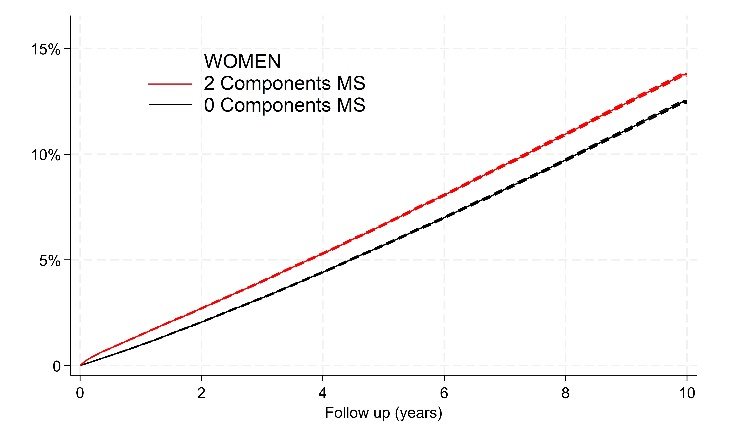

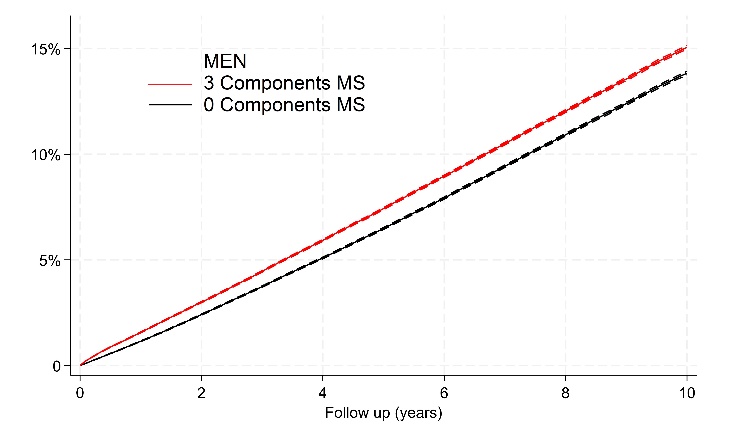

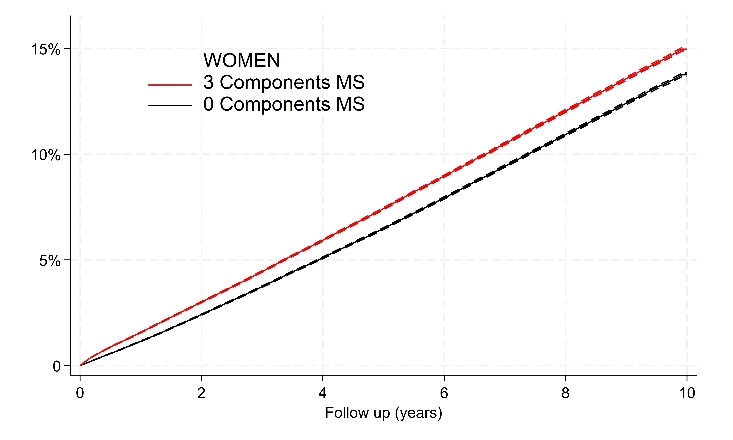

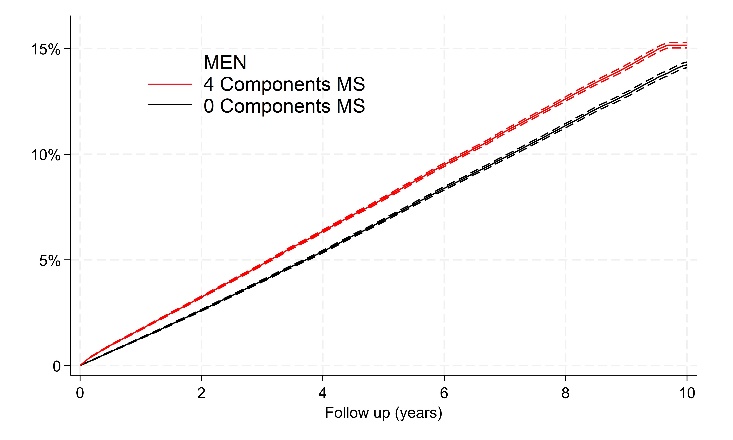

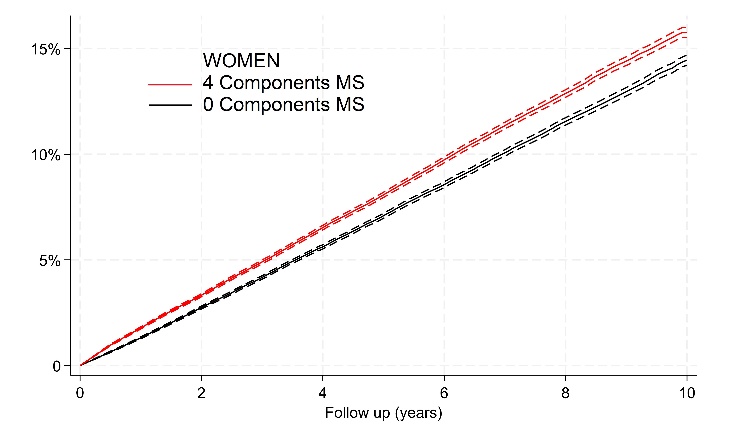

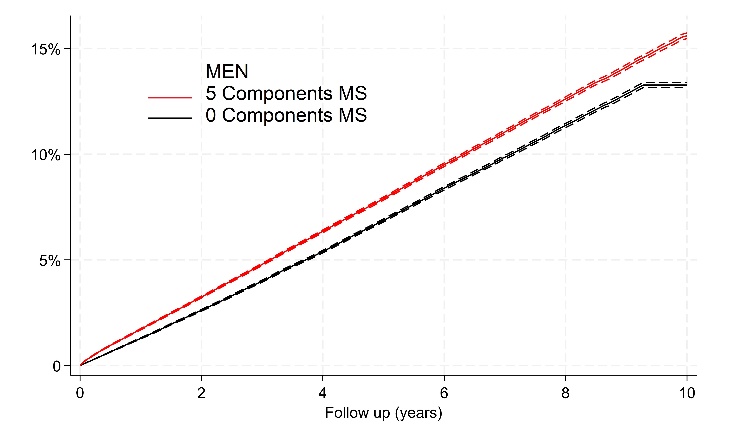

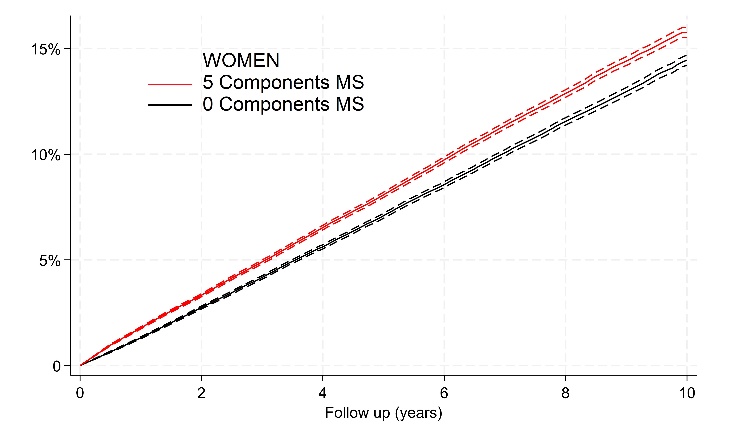


**Supplementary Figure 19.** The combined effect of MS components on overall cancer incidence compared with individuals without any MS component (without skin non melonoma cancer)


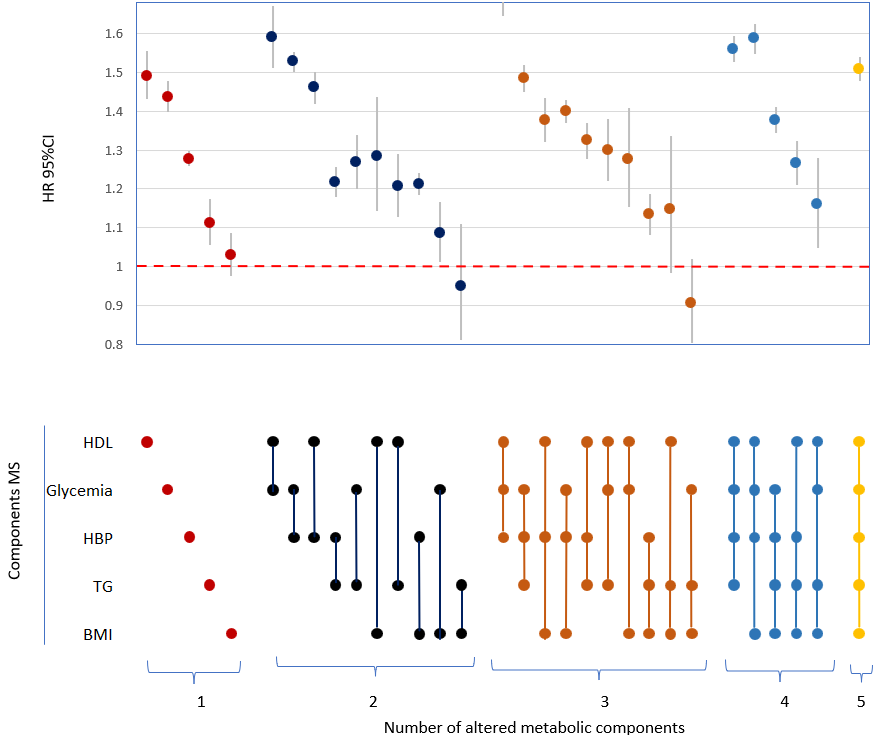


HRs arepresented by circles, with their 95% CIs as vertical lines; HR, hazard ratio; CI, confidence interval. Reference category is 0 components. Cox models adjusted by age, MEDEA Deprivation Index, smoking status and nationality.

**Supplementary Figure 20.** The combined effect of components of MS on overall cancer incidence. Stratified by age


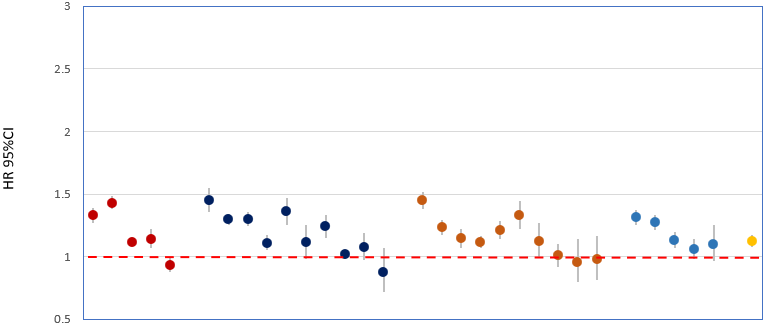


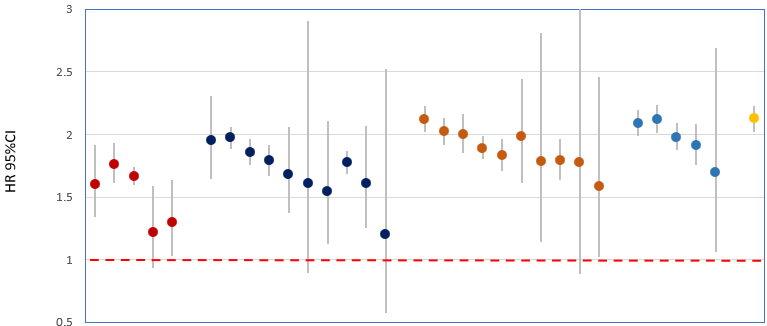

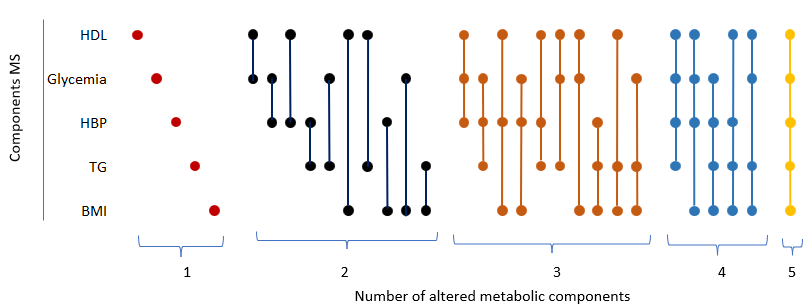

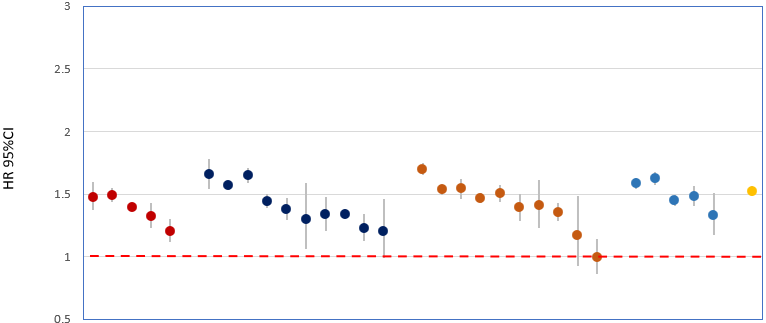


40-59 years

80-100 years

60-79 years
